# IL-2–mTORC1 signaling coordinates the STAT1/T-bet axis to ensure Th1 cell differentiation and anti-bacterial immune response in fish

**DOI:** 10.1371/journal.ppat.1010913

**Published:** 2022-10-25

**Authors:** Kete Ai, Kang Li, Xinying Jiao, Yu Zhang, Jiaqi Li, Qian Zhang, Xiumei Wei, Jialong Yang

**Affiliations:** 1 State Key Laboratory of Estuarine and Coastal Research, School of Life Sciences, East China Normal University, Shanghai, China; 2 Laboratory for Marine Biology and Biotechnology, Qingdao National Laboratory for Marine Science and Technology, Qingdao, China; Duke University, UNITED STATES

## Abstract

Utilization of specialized Th1 cells to resist intracellular pathogenic infection represents an important innovation of adaptive immunity. Although transcriptional evidence indicates the potential presence of Th1-like cells in some fish species, the existence of CD3^+^CD4^+^IFN-γ^+^ T cells, their detailed functions, and the mechanism determining their differentiation in these early vertebrates remain unclear. In the present study, we identified a population of CD3^+^CD4-1^+^IFN-γ^+^ (Th1) cells in Nile tilapia upon T-cell activation *in vitro* or *Edwardsiella piscicida* infection *in vivo*. By depleting CD4-1^+^ T cells or blocking IFN-γ, Th1 cells and their produced IFN-γ were found to be essential for tilapia to activate macrophages and resist the *E*. *piscicida* infection. Mechanistically, activated T cells of tilapia produce IL-2, which enhances the STAT5 and mTORC1 signaling that in turn trigger the STAT1/T-bet axis-controlled IFN-γ transcription and Th1 cell development. Additionally, mTORC1 regulates the differentiation of these cells by promoting the proliferation of CD3^+^CD4-1^+^ T cells. Moreover, IFN-γ binds to its receptors IFNγR1 and IFNγR2 and further initiates a STAT1/T-bet axis-mediated positive feedback loop to stabilize the Th1 cell polarization in tilapia. These findings demonstrate that, prior to the emergence of tetrapods, the bony fish Nile tilapia had already evolved Th1 cells to fight intracellular bacterial infection, and support the notion that IL-2–mTORC1 signaling coordinates the STAT1/T-bet axis to determine Th1 cell fate, which is an ancient mechanism that has been programmed early during vertebrate evolution. Our study is expected to provide novel perspectives into the evolution of adaptive immunity.

## Author summary

Differentiation of T helper (Th) cells provides the immune system with specialized T-cell subsets, which ensures a more effective immune response and reduces energy expenditure, thus representing a key innovation of adaptive immunity. Although leukocytes, CD4-1^+^ lymphocytes, or CD3^+^ T cells of some bony fish have been reported to express IFN-γ at the transcriptional level, indicating that Th1-like cells exist in these early vertebrates, the CD3^+^CD4-1^+^IFN-γ^+^ T-cell population has not been identified in cold-blooded animals. Here, we demonstrated the existence of CD3^+^CD4-1^+^IFN-γ^+^ Th1 cells in Nile tilapia, explored their immunological function in resisting *Edwardsiella piscicida* infection, and investigated their differentiation mechanisms. Our findings suggest that prior to the emergence of tetrapods, Nile tilapia already utilized well-evolved Th1 cells to fight intracellular bacterial infection and that the mechanisms underpinning Th1 cell differentiation in mammals have been programmed early during vertebrate evolution.

## Introduction

CD4^+^ T cells in the adaptive immune system had long been neglected owing to their inability to directly kill infected cells or neutralize pathogens. However, with increasing research, this population of T cells was found to be able to recruit and activate other immune cells, enhance their immunological activity, orchestrate immune responses, and resist pathogen infection by producing multiple cytokines and chemokines, thus playing a central role in adaptive immunity [1]. Moreover, studies focusing on the differentiation of CD4^+^ T cells have suggested that these lineages display the hallmarks of adaptive immunity and are indispensable [2]. Upon pathogen infection, the functional specialization of CD4^+^ T cells is determined according to the antigen signals and microenvironmental cues [3]. For example, viruses and intracellular bacteria trigger differentiation of CD4^+^ T cells into Th1 cells, which activate macrophages and cytotoxic T cells by secreting interferon (IFN)-γ, lymphotoxin (LT)-α, and interleukin (IL)-2 and consequently contribute to infection elimination [4]. In contrast, CD4^+^ T cells selectively differentiate into IL-4-producing Th2 cells or IL-17-producing Th17 cells to resist infections caused by extracellular parasites or bacteria [5]. Another specialized CD4^+^ T cell subset, Treg, suppresses the T-cell response and prevents the occurrence of autoimmune diseases [6]. Thus, the CD4^+^ T cells differentiate into several subsets to exert specific functions, which increases the efficiency but limits the scale of the immune response and energy expenditure, ensuring the optimal immune response against a particular pathogen.

Th cell fate determination is not a simple on/off switch or accomplished in a single step; it is a highly sophisticated process jointly regulated by T-cell receptor (TCR) signaling, cytokines, epigenetic modifications, chromatin remodeling, and metabolic reprogramming [1,7]. The Th1 subset is the first identified Th cell lineage, whose differentiation is induced by the cytokines IL-2, IL-12, and IFN-γ. By binding to their receptors on CD4^+^ T cells, these cytokines activate STAT1 and STAT4, which in turn promote the expression of the key transcription factor T-bet and consequently drive the Th1 cell differentiation [8,9]. The developed Th1 cells release a large amount of IFN-γ, which promotes Th1 cell commitment through a positive feedback pathway, inducing them to become highly polarized and further stabilizing the differentiation [10]. In addition to the cytokines and transcription factors, signals downstream of the TCR, for example Ca^2+^-NFAT, MAPK, and mTORC1 signaling, are also of paramount importance for Th1 cell differentiation [11,12]. However, our knowledge about Th1 cell differentiation is mainly derived from mammalian models (e.g., mouse and human); the immunological functions of Th1 or Th1-like cells in early vertebrates and the mechanisms underpinning their differentiation remain largely unclear.

Bony fish are a group of early vertebrates with a classical adaptive immune system and primordial T cells, and they are therefore considered as ideal models for studying the evolution of T-cell immunity [13]. In the past two decades, several subpopulations of T cells (i.e., CD8^+^, CD4-1^+^, CD4-2^+^, and TCRγδ^+^ T cells) have been identified in various fish species such as carp, rainbow trout, pufferfish, and zebrafish; their immunological functions regarding cytotoxicity, resistance to bacterial infection and allogeneic cell transfer, or antigen presentation have also been preliminarily confirmed [14–23]. These findings suggest that prior to the emergence of tetrapods, primordial T cells in bony fish were already equipped with sophisticated immunological functions. It has been established that except for a few fish species (i.e., anglerfish and cod) that have lost the CD4/MHCII axis during evolution [24,25], most bony fish possess a CD4^+^ T-cell population. The fact that leukocytes or sorted CD4-1^+^ lymphocytes from several bony fish species express the IFN-γ, IL-4, and IL-17 genes and the transcription factors T-bet, GATA3, and RORγt, indicates that Th1-, Th2- and Th-17-like CD4^+^ T cells might exist in fish species [26–32]. However, despite these advances, the existence of CD3^+^CD4^+^IFN-γ^+^ T cells, their detailed immunological functions, and differentiation mechanisms have not been well elucidated in bony fish. Recently, using a Nile tilapia (*Oreochromis niloticus*) model, we demonstrated that several classical pathways, such as Ca^2+^-NFAT, MAPK/ERK, NF-κB, and mTORC1 signaling, are indispensable for the activation and proliferation of T cells and their role in fighting bacterial infection [33–36]. In the present study, we identified a population of CD3^+^CD4-1^+^IFN-γ^+^ T cells (designated Th1 cells) in Nile tilapia using mAbs generated against CD3ε, CD4-1, and IFN-γ, and further investigated their immunological roles in resisting intracellular bacterial infection and the mechanism underlying their potential differentiation. Our results suggest that the bony fish Nile tilapia has evolved Th1 cells to fight intracellular bacterial infection and that the mechanisms underpinning Th1 cell differentiation in modern mammals had been programmed before the emergence of tetrapods.

## Results

### Activated T cells of tilapia produce IFN-γ

**Fig 1:**
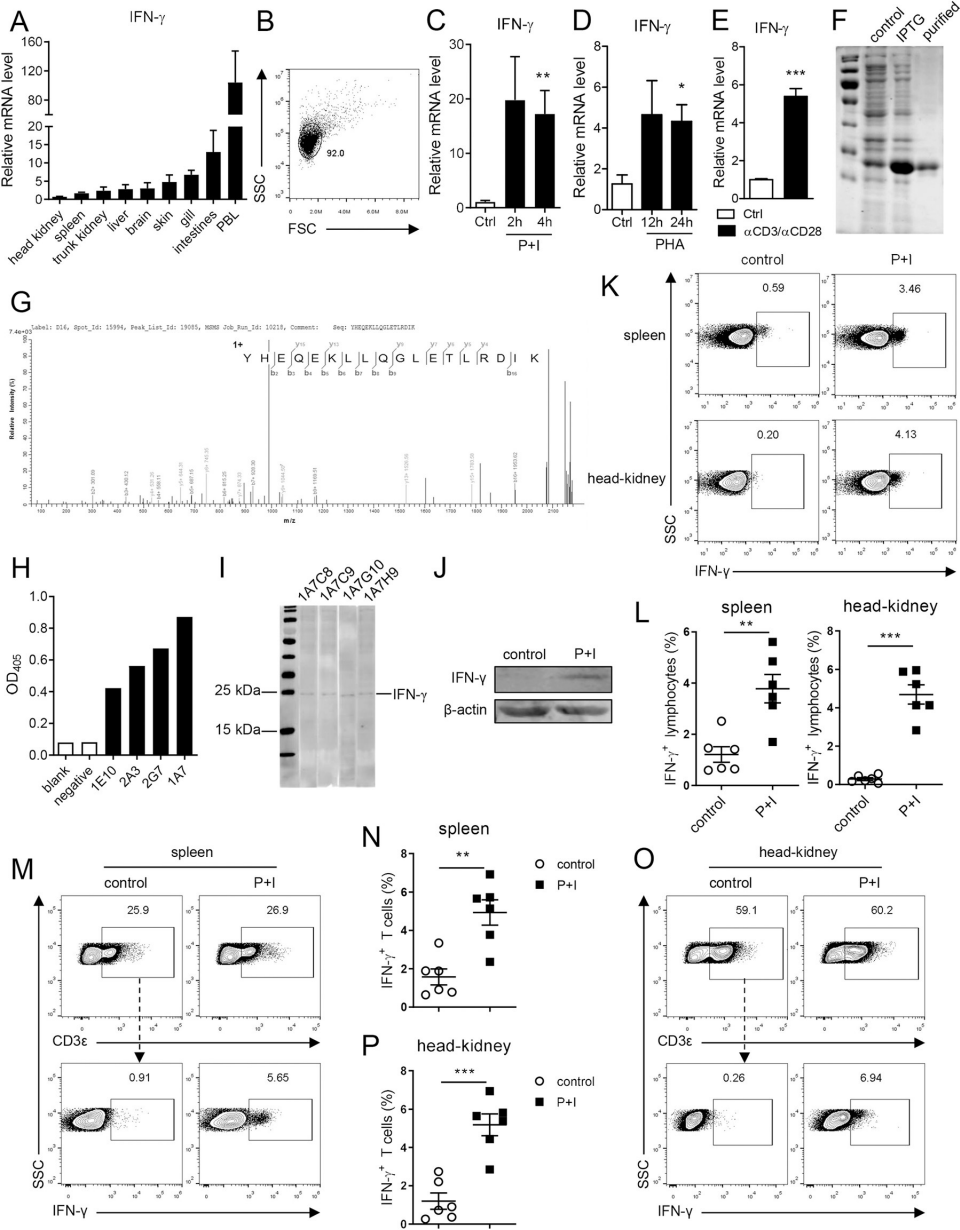
Tilapia T cells produce IFN-γ. (**A**) The expression of IFN-γ in different tissues was examined by qPCR, n = 5. (**B**) Flow cytometry analyzed the FSC/SSC profiles of spleen leukocytes and the gate represents lymphocytes. (**C**-**E**) Spleen leukocytes were stimulated with PMA plus ionomycin (P+I) (**C**), PHA (**D**) or α-CD3ε/α-CD28 (**E**), and relative mRNA levels of IFN-γ were examined by qPCR, n = 4–6. (**F**) SDS-PAGE assay of recombinant IFN-γ protein. (**G**) Maldi-TOF-TOF analysis of recombinant IFN-γ protein. (**H**) Candidate IFN-γ mAbs were screened by ELISA. (**I**) Western blot assay showed the binding of mAbs to overexpressed tilapia IFN-γ in HEK 293T cells. (**J-P**) Spleen or head kidney leukocytes were stimulated with P+I for 4 h in the presence of GolgiPlug. The expression of IFN-γ in leukocytes was detected by western blot (**J**) or flow cytometry (**K**), and the expression of IFN-γ in CD3^+^ T cells from spleen (**M**) and head kidney (**O**) were detected by flow cytometry. Scatter plot figures showed the percentages of IFN-γ in gated lymphocytes (**L**) or CD3^+^ T cells (**N, P**), n = 6. These experiments were repeated for three independent times. *: *p*<0.05, **: *p*<0.01, ***: *p*<0.001, determined by a two-tailed Student’s t-test.

The cloning of Nile tilapia IFN-γ cDNA has been reported in a previous study [37]. In the present study, we found that the IFN-γ gene is located on chromosome LG17 of Nile tilapia and its gene location and structure are evolutionarily conserved, as revealed by the synteny and exon–intron analyses (S1A and S1B Fig). As previously shown tilapia IFN-γ possesses a signature motif and nuclear localization signal (NLS), and it clusters well with IFN-γ homologues from other vertebrates (S1C and S1D Fig). These observations thus suggest that the Nile tilapia encodes a typical IFN-γ. As a multipotent cytokine, IFN-γ is principally produced by lymphocyte lineages such as Th1 cells, CD8^+^ T cells, and natural killer cells. Here, IFN-γ transcription was detected in all examined lymphoid-related tissues of tilapia, including head kidney, spleen, trunk kidney, liver, brain, skin, gill, intestine, and peripheral blood, with the highest expression level in peripheral blood (Fig 1A). To investigate the association of IFN-γ with lymphocytes in tilapia, we used a previously established protocol to isolate tilapia leukocytes. According to our previous report [36], lymphocytes constitute more than 90% of the isolated spleen leukocytes (Fig 1B). Thus, these spleen leukocytes containing a high proportion of lymphocytes were considered as lymphocytes in subsequent experiments.

IFN-γ transcription in spleen lymphocytes was markedly induced by PMA plus ionomycin (P+I) stimulation (Fig 1C), indicating that activated lymphocytes potentially produce IFN-γ in tilapia. To further assess the expression of IFN-γ by T-cell lineages, spleen lymphocytes were stimulated by T-cell specific mitogen PHA or anti-tilapia CD3ε mAb plus anti-tilapia CD28 mAb. As predicted, T-cell activation enhanced IFN-γ expression (Fig 1D and 1E), thus providing transcriptional evidence that activated T cells of tilapia produce IFN-γ. However, evidence regarding IFN-γ-producing cells in early vertebrates is still lacking at the protein level owing to the scarcity of reliable antibodies. To this end, tilapia IFN-γ recombinant protein was prepared using a prokaryotic expression system (Fig 1F), confirmed by mass spectrometry (Fig 1G), and used to immunize mice for mAb development. After four rounds of immunizations, the splenocytes of mice were fused with SP2/0 myeloma cells. The supernatants from more than ten hybridoma clones, including 1E10, 2A3, 2G7, and 1A7, showed binding to recombinant tilapia IFN-γ (Fig 1H). The hybridoma 1A7 was then selected for sub-cloning by limiting dilution, and supernatants from sub-clone 1A7C8, 1A7C9, 1A7G10, and 1A7H9 were confirmed to specifically recognize overexpressed tilapia IFN-γ in HEK 293T cells (Fig 1I). Hybridoma cells obtained from the clone 1A7H9 were intraperitoneally injected into mice, and ascites were collected, followed by purification and biotin labelling of mAbs. Using this IFN-γ mAb, we revealed that spleen lymphocytes produced IFN-γ protein upon activation by P+I stimulation (Fig 1J). In addition, the proportion of IFN-γ^+^ lymphocytes in spleen and head kidney leukocytes was markedly increased after activation (Fig 1K and 1L). Furthermore, using tilapia CD3ε mAb, we found that the percentage of CD3^+^ T cells that produced IFN-γ was significantly higher after P+I stimulation in both spleen (Fig 1M and 1N) and head kidney (Fig 1O and 1P), suggesting that activated T cells of tilapia produce IFN-γ. Therefore, our findings at the transcriptional, protein, and cellular levels collectively support the conclusion that activated T cells of Nile tilapia produce the cytokine IFN-γ.

### Th1 cells are present in Nile tilapia

**Fig 2:**
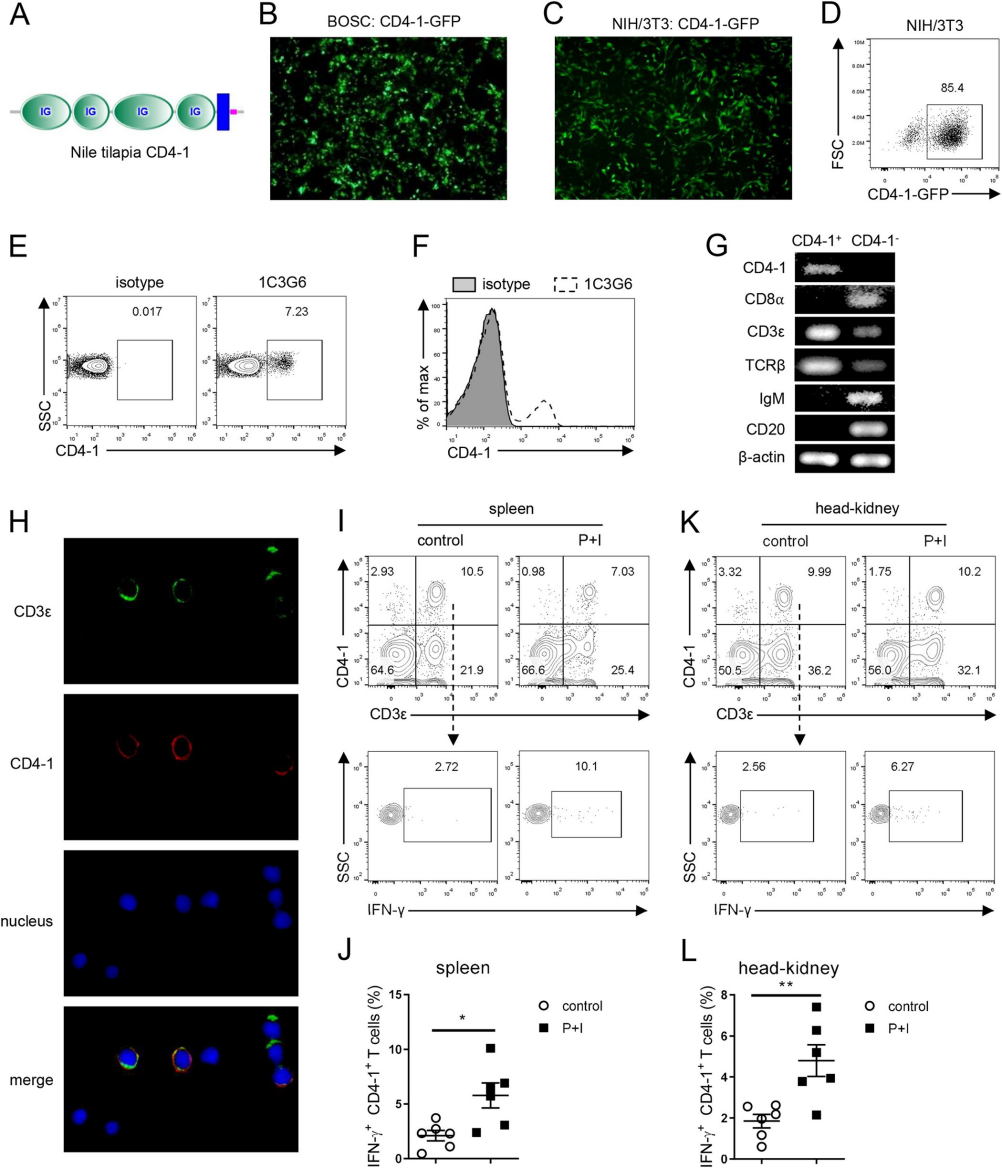
Th1 cells are present in tilapia. (**A**) Domain prediction of tilapia CD4-1. The accession number of CD4-1 was listed in S1 Table. (**B**) BOSC23 cells were transfected with MIGR1-CD4-1 to produce retrovirus, and the fluorescence microscopy showed the BOSC23 cells expressing CD4-1. (**C**, **D**) Fluorescence microscopy (**C**) and flow cytometry (**D**) showed the NIH/3T3 cells expressing tilapia CD4-1. (**E**, **F**) Dot plots (**E**) and overlaid histogram (**F**) showed the tilapia spleen leukocytes stained with the CD4-1 mAb 1C3G6. (**G**) Gene expression profiles of CD4-1^+^ and CD4-1^-^ lymphocytes were detected by RT-PCR. (**H**) Immunofluorescence showed spleen leukocytes that stained with CD3ε (green) and CD4-1 (red) mAbs. (**I-L**) Spleen and head kidney leukocytes were stimulated with P+I for 4 h in the presence of GolgiPlug. Flow cytometry showed the expression of IFN-γ in CD3^+^CD4-1^+^ T cells from spleen (**I**) and head kidney (**K**). The percentages of IFN-γ in CD3^+^CD4-1^+^ T cells from spleen (**J**) and head kidney (**L**), n = 6. These experiments were repeated for three independent times. *: *p*<0.05, **: *p*<0.01, ***: *p*<0.001, determined by a two-tailed Student’s t-test.

IFN-γ is a signature cytokine of Th1 cells. Although sorted CD4-1^+^ lymphocytes of zebrafish have been reported to express IFN-γ at the mRNA level [32], evidence at the protein level and for CD3^+^CD4-1^+^ T cells is still lacking. Therefore, we sought to investigate the presence of CD3^+^CD4^+^IFN-γ^+^ cells in tilapia. In bony fish, the CD4-1 receptor with four Ig domains is considered as a homologue of mammalian CD4 [14]. Herein, we developed CD4-1 mAb to identify the CD4^+^ T-cell lineage in tilapia. The full-length coding region of CD4-1, which encodes four Ig domains and one transmembrane domain (Fig 2A), was cloned into the MIGR1 vector. Then, BOSC23 packaging cells were transfected with the resulting plasmid for retrovirus generation (Fig 2B). NIH/3T3 cells were infected with the CD4-1 retrovirus to obtain CD4-1-expresssing cells, which were used for mouse immunization (Fig 2C and 2D). After four immunizations, mouse splenocytes were fused with SP2/0 myeloma cells, and the hybridomas were screened by flow cytometry. The supernatant of the hybridoma 1C3G6 yielded a distinct positive population among gated tilapia spleen lymphocytes (Fig 2E and 2F). To determine whether this candidate mAb recognizes the tilapia CD4-1 receptor, we sorted the CD4-1^+^ or CD4-1^-^ spleen lymphocytes and analyzed their gene expression patterns. CD4-1 was expressed specifically in CD4-1^+^ lymphocytes, whereas CD8α, IgM, and CD20 were expressed only in CD4-1^-^ cells (Fig 2G). Additionally, CD4-1^+^ lymphocytes showed considerably higher transcript levels of CD3ε and TCRβ than CD4-1^-^ lymphocytes (Fig 2G). Furthermore, the immunofluorescence staining of spleen lymphocytes using anti-CD3ε and anti-CD4-1 mAbs showed that most CD4-1^+^ cells were CD3ε positive, whereas some CD3ε^+^ cells, likely CD8^+^ T cells, did not express CD4-1 (Fig 2H). These findings collectively confirmed the validity of this CD4-1 mAb, and the availability of reliable mAbs against CD3ε, CD4-1, and IFN-γ facilitates the study of Th1 cell response in tilapia. Flow cytometry revealed that, within the gated spleen lymphocyte population, few CD3^+^CD4-1^+^ T cells were IFN-γ^+^ in absence of stimulation (Fig 2I and 2J). However, activation of T cells by P+I markedly enhanced the capacity of CD3^+^CD4-1^+^ T cells to produce IFN-γ, as indicated by the higher proportion of CD3^+^CD4-1^+^IFN-γ^+^ T cells (Fig 2I and 2J). Similar results were found in the head kidney of Nile tilapia (Fig 2K and 2L). Overall, these results indicate for the first time that CD3^+^CD4-1^+^IFN-γ^+^ Th1 cells are present in bony fish.

### Th1 cells are crucial for tilapia to resist intracellular bacterial infection

**Fig 3:**
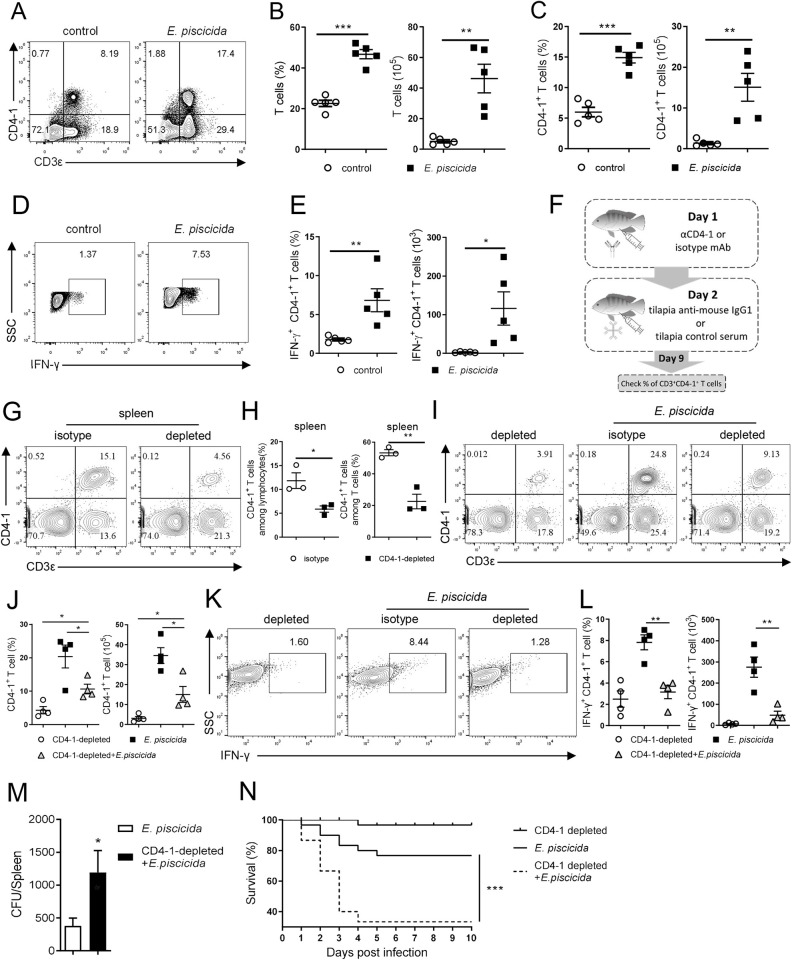
Tilapia Th1 cells are essential for resisting intracellular bacterial infection. **(A-E)** Tilapia individuals that infected with *E*. *piscicida* or uninfected controls were *i*.*p*. injected with BFA 6 hours before sacrifice, and spleen lymphocytes were isolated for assays. **(A)** Flow cytometry showed the cells stained with CD3ε and CD4-1 mAbs. (**B, C**) Scatter plot figures showed the percentage and absolute numbers of CD3^+^ T cells **(B)** and CD3^+^CD4-1^+^ T cells **(C)**, n = 5. (**D, E**) Representative dot plots (**D**), percentage and absolute numbers (**E**) of IFN-γ^+^ cells in gated CD3^+^CD4-1^+^ T cells, n = 5. **(F)** Strategy for CD4-1^+^ cells depletion. **(G)** Flow cytometry showed the percentage of CD3^+^CD4-1^+^ T cells in spleen lymphocytes of CD4-1-depleted or non-depleted tilapia on 9-day post depletion. **(H)** Scatter plot figures showed the percentages of CD4-1^+^ T cells among lymphocytes or T cells of isotype control and CD4-1-depleted tilapia, n = 3. **(I-N)** Tilapia individuals were infected with *E*. *piscicida* or not on day 9 after depletion, and the spleen leukocytes were harvested on 5 DPI for assay. Flow cytometry and scatter plot figures showed the percentage and absolute numbers of CD3^+^CD4-1^+^ T cells **(I, J)** and CD3^+^CD4-1^+^IFN-γ^+^ T cells **(K, L)**, n = 4. **(M)** *E*. *piscicida* titers in spleen of CD4-1-depleted or nondepleted tilapia on 5 DPI, n = 5. **(N)** Kaplan-Meyer survival plot showed the survival percentage of CD4-1-depleted or nondepleted tilapia during *E*. *piscicida* infection, n = 25. These experiments were repeated for at least two independent times. *: *p*<0.05, **: *p*<0.01, ***: *p*<0.001, determined by a two-tailed Student’s t-test.

Next, we investigated whether Th1 cells of tilapia exert immunological functions. In mammals, upon encountering an intracellular pathogen infection, naïve CD4^+^ T cells preferentially differentiate into Th1 cells, which predominantly produce IFN-γ and lymphotoxin, leading to the eradication of intracellular pathogens [1]. However, the involvement of Th1 cells in the adaptive immune response and their detailed functions in bony fish are not well understood. In the present study, the Th1 cell response was investigated after tilapia was infected by the intracellular bacteria *Edwardsiella piscicida*. On 5 days post infection (DPI), the percentage and absolute number of CD3^+^ splenic T cells were markedly higher than those in the control fish (Fig 3A and 3B), indicating the robust initiation of a T-cell response during the primary immune response in tilapia. Moreover, CD3^+^CD4-1^+^ T cells were more abundant in tilapia infected with *E*. *piscicida* than in control one (Fig 3A and 3C). Notably, more CD3^+^CD4-1^+^ T cells were observed to produce IFN-γ in infected fish (Fig 3D and 3E), suggesting that intracellular bacterial infection triggers a robust Th1 cell response in Nile tilapia. To further verify the crucial role of CD4^+^ T cells or Th1 cells in anti-bacterial immune response, CD4-1^+^ cells were selectively depleted in tilapia using anti-CD4-1 mAb in combination with tilapia anti-mouse IgG1 serum (Fig 3F). On day 9 after antibody administration, approximately 60% of CD4-1^+^ T cells were depleted in the experimental group (Figs 3G and 3H and S2). Then, the animals were infected with *E*. *piscicida*. Compared with the uninfected tilapia (Fig 3A and 3B), nondepleted fish infected with *E*. *piscicida* showed a marked expansion of CD4-1^+^ T cells on 5 DPI (Fig 3I middle panel and 3J), but infected fish with CD4-1^+^ T-cell depletion exhibited a significant defect in CD4-1^+^ T-cell expansion during bacterial infection (Fig 3I and 3J). Notably, the ability of CD3^+^CD4-1^+^ T cells to produce IFN-γ (Fig 3K) and the percentage and absolute number of CD3^+^CD4-1^+^IFN-γ^+^ Th1 cells (Fig 3L) were markedly lower in CD4-1^+^ T-cell-depleted tilapia. The deficiency of CD3^+^CD4-1^+^ T cells and Th1 cells made it challenging to manage the infection (Fig 3M) and rendered the tilapia more vulnerable to *E*. *piscicida* (Fig 3N). Collectively, our findings suggest that Th1 cells are crucial for tilapia to resist intracellular bacterial infection.

### Th1 cells facilitate the anti-bacterial response of tilapia by enhancing macrophage activity

**Fig 4:**
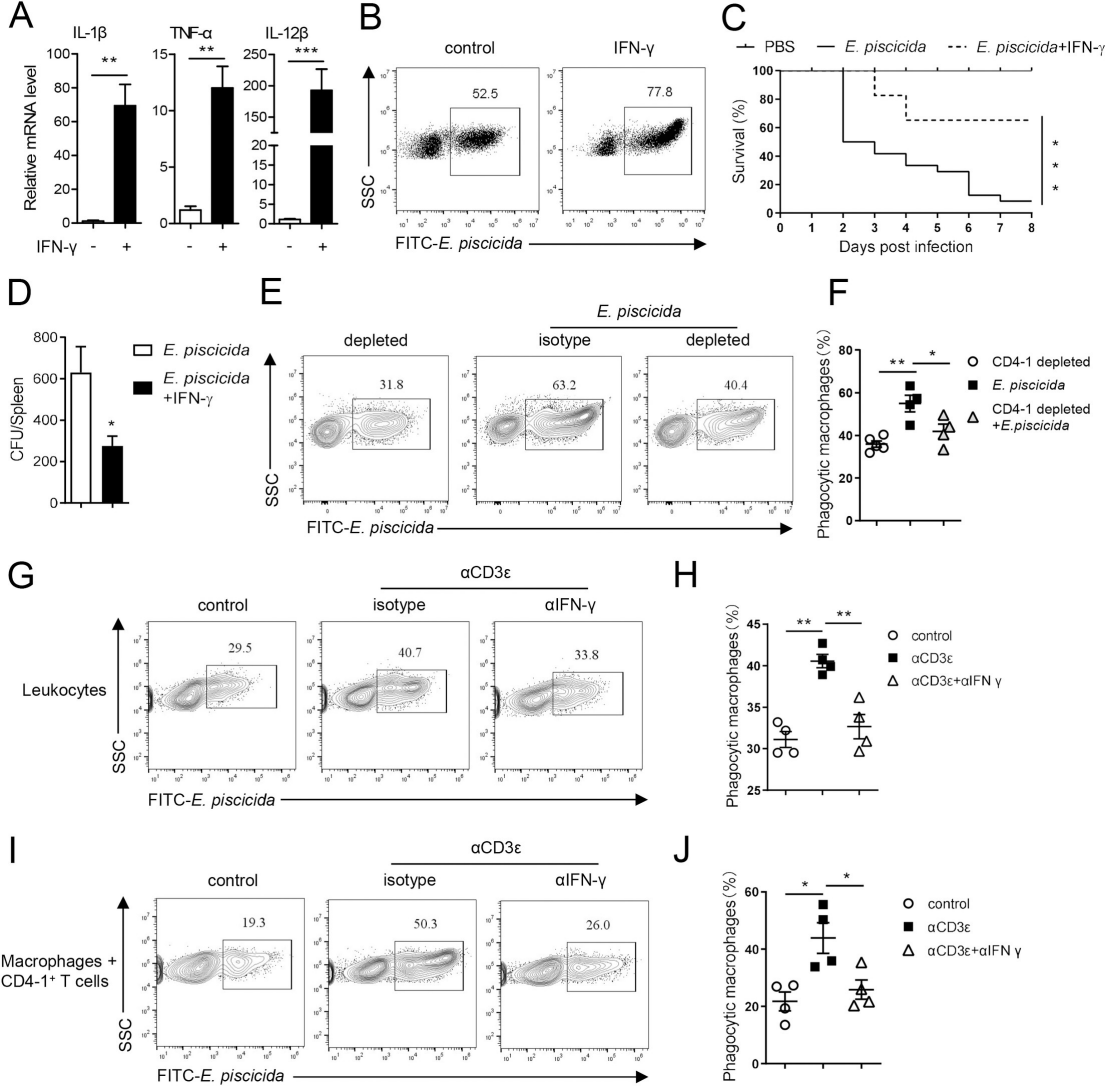
Th1 cells activate macrophages to facilitate anti-bacterial immune response in tilapia. (**A**) Head kidney macrophages were stimulated with recombinant IFN-γ protein, and relative mRNA levels of indicated molecules were examined at 12 h post stimulation by qPCR, n = 4–6. (**B**) Dot plots showed the phagocytic rates of head kidney macrophages against FITC-labelled *E*. *piscicida* in the presence or absence of recombinant IFN-γ. (**C, D**) Tilapia individuals that infected with *E*. *piscicida* were injected with recombinant IFN-γ or PBS. (**C**) Kaplan-Meyer survival plot showed the survival percentage of tilapia, n = 25. (**D**) *E*. *piscicida* titers in spleen on 5 DPI, n = 6. (**E, F**) CD4-1^+^ cells were depleted as described in Fig 3F. Tilapia individuals were infected with *E*. *piscicida* on day 9 after depletion, and head kidney leukocytes were harvested on 5 DPI for assay. The flow cytometry (**E**) and scatter plot figures (**F**) showed the phagocytosis of macrophages from CD4-1-depleted or nondepleted tilapia against FITC-labelled *E*. *piscicida* at 12 h, n = 4–5. (**G, H**) The head kidney leukocytes that stimulated with CD3ε mAb or not in the presence of IFN-γ mAb or isotype antibody were incubated with FITC-labelled *E*. *piscicida*. The flow cytometry (**G**) and scatter plot figures (**H**) showed the phagocytosis of macrophages at 12 h, n = 4. (**I, J**) The head kidney macrophages and CD3^+^CD4-1^+^ T cells that sorted from the same tilapia individual were co-cultured with or without CD3ε mAb, IFN-γ mAb and FITC-labelled *E*. *piscicida*. The flow cytometry (**I**) and scatter plot figures (**J**) showed the phagocytosis of macrophages at 12 h, n = 4. These experiments were repeated for at least two independent times. *: *p*<0.05, **: *p*<0.01, ***: *p*<0.001, determined by a two-tailed Student’s t-test.

Because Th1 cells were involved in resisting the *E*. *piscicida* infection in tilapia, we further investigated the precise mechanism by which Th1 cells mediated this immune response. First, we found that recombinant IFN-γ activated tilapia macrophages *in vitro*, as evidenced by the induced expression of proinflammatory cytokines IL-1β, TNF-α, and IL-12 in these cells (Fig 4A), and consequently enhanced their phagocytic ability against *E*. *piscicida* (Fig 4B). *E*. *piscicida* infection caused severe mortality in tilapia within 8 days (Fig 4C), but IFN-γ supplementation rendered the animals less vulnerable to this pathogen (Fig 4C) and promoted elimination of the infection, as indicated by the lower bacterial burden in the spleen (Fig 4D). Moreover, in CD4-1^+^ cell-depleted tilapia, whose Th1 cells were markedly impaired during *E*. *piscicida* infection (Fig 3K and 3L), the phagocytic ability of macrophages was correspondingly weakened (Fig 4E and 4F), indicating the critical role of CD4^+^ T cells or Th1 cells in regulating macrophage activity in tilapia. The dependency of macrophage activity on activated T cells was also confirmed by an *in vitro* experiment, in which macrophages in CD3-mAb-activated leukocytes exhibited higher phagocytic activity toward *E*. *piscicida* (Fig 4G and 4H). However, the increase in phagocytic activity induced by activated T cells was markedly blocked by anti-IFN-γ mAb (Fig 4G and 4H), suggesting that T cells facilitate macrophage-mediated immune response by producing IFN-γ. To further confirm whether the IFN-γ secreted by Th1 cells enhances macrophage anti-bacterial activity in a cognate manner, we sorted macrophages and CD3^+^CD4-1^+^ T cells from a single tilapia individual. The macrophages co-cultured with activated CD4^+^ T cells exhibited robust phagocytic activity toward *E*. *piscicida*, and this response was significantly diminished by adding anti-IFN-γ mAb (Fig 4I and 4J), indicating the essential role of IFN-γ produced by CD3^+^CD4-1^+^ T cells in regulating macrophage activity. Thus, these data demonstrate that Th1 cells facilitate the anti-bacterial response of tilapia by enhancing macrophage activity.

### Tilapia utilize the conserved STAT1/T-bet axis to initiate Th1 cell differentiation

The IFN-γ production and Th1 cell differentiation are transcriptionally controlled by multiple transcription factors. Of these, T-bet, STAT1, and STAT4 are the most important factors responsible for Th1 cell development [38], and we found that all these transcription factors are encoded in the Nile tilapia genome (S3A Fig). Similar to its mouse homologue, tilapia T-bet possesses the TBOX domain, a conserved core domain for sequence-specific DNA binding (S3B Fig). Additionally, arrangement of the functional domains of tilapia STAT1 and STAT4 is similar to that in their respective mouse homologues (S3C and S3D Fig). Both STAT1 and STAT4 proteins contain a STAT_int domain at the N terminus, which is crucial for protein interaction, phosphorylation, nuclear translocation, and transcriptional activity [39,40]. The STAT_int domains are followed by a STAT_α domain, a STAT_bind domain, and an SH2 domain, which ensure the proper interaction of STAT1/4 with proteins, DNA, and the phosphorylated tail of other STATs, respectively [41–43]. A STAT1 TAZ2-binding domain, which enables a protein to induce or modulate the transcription of target genes [44], is located at the C-terminal end of STAT1 but not STAT4 (S3C and S3D Fig). Notably, the tertiary structure of tilapia T-bet, STAT1, and STAT4, was highly similar to that of their respective mouse homologues (S3E–S3G Fig). Together, these observations suggest that tilapia possesses a set of evolutionarily conserved transcription factors for Th1 cell differentiation.

**Fig 5:**
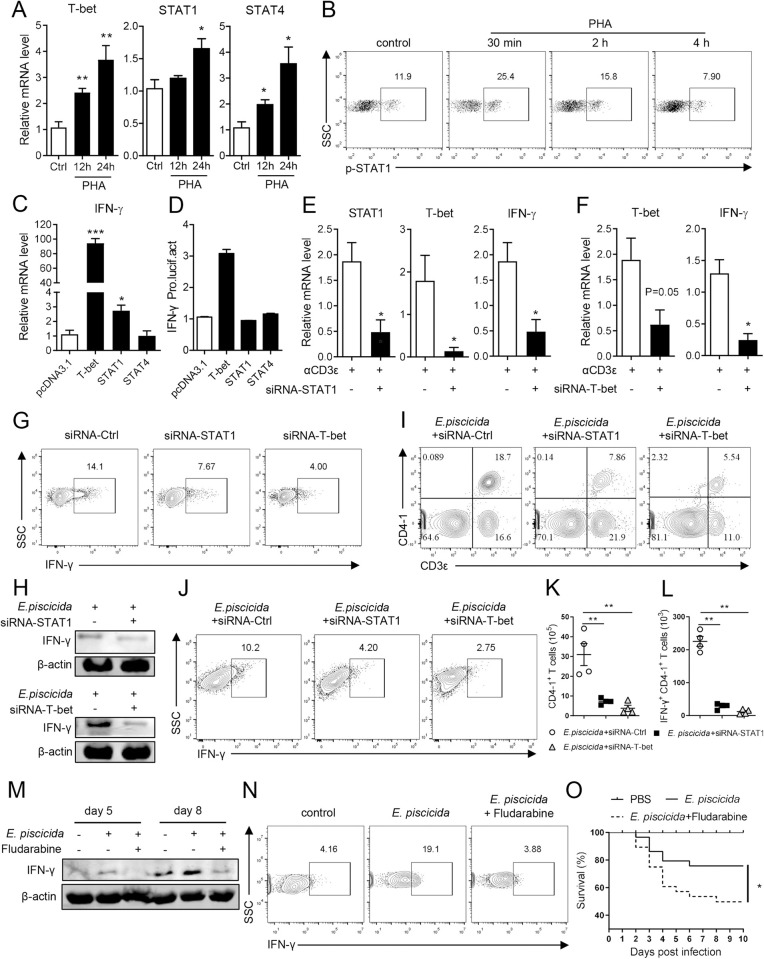
STAT1/T-bet axis regulates the Th1 differentiation in tilapia. (**A**) Spleen leukocytes were stimulated with PHA for 12 h. Relative mRNA levels of T-bet, STAT1 and STAT4 were examined by qPCR, n = 4. (**B**) Flow cytometry showed the phosphorylation level of STAT1 in lymphocytes that stimulated with PHA. (**C**) Transcriptional levels of IFN-γ in HEK 293T cells that transfected with tilapia T-bet, STAT1 and STAT4, n = 6. (**D**) HEK 293T cells were co-transfected with tilapia T-bet, STAT1 or STAT4, and pGL3-IFN-γ promoter. The LUC activities were assessed at 48 h post-transfection, n = 4. (**E-G**) Spleen leukocytes from tilapia that *i*.*p*. injected with T-bet-specific, STAT1-specific or control siRNA for 2 days were harvested and stimulated with CD3ε mAb for 12 h. Relative mRNA levels of STAT1, T-bet and IFN-γ (**E, F**, n = 4), and the percentage of CD3^+^CD4-1^+^IFN-γ^+^ T cells (**G**) were examined. (**H-L**) Tilapia *i*.*p*. injected with T-bet-specific, STAT1-specific or control siRNA were infected with *E*. *piscicida*. Tilapia was *i*.*p*. injected with BFA 6 h before sacrifice, and spleen leukocytes were harvest for assay. Western blot assay showed the expression of IFN-γ at 48 h post infection (**H**). Flow cytometry and scatter plot figures showed the percentage and absolute numbers of CD3^+^CD4-1^+^ T cells **(I, K)** and CD3^+^CD4-1^+^IFN-γ^+^ T cells **(J, L)** on 5 DPI, n = 4. (**M-O**) Tilapia individuals that infected with *E*. *piscicida* were injected with STAT1 inhibitor Fludarabine or PBS, and animals were *i*.*p*. injected with BFA 6 hours before sacrifice. (**M**) Western blot assay showed the protein levels of IFN-γ in spleen leukocytes on indicated days. (**N**) Flow cytometry showed the percentage of IFN-γ in spleen CD4-1^+^ T cells on day 7 post-infection. (**O**) Kaplan-Meyer survival plot showed the survival percentage of tilapia, n = 25. These experiments were repeated for at least two independent times. *: *p*<0.05, **: *p*<0.01, ***: *p*<0.001, determined by a two-tailed Student’s t-test. The accession numbers of selected sequences were listed in S1 Table.

Next, we examined the regulation of these transcription factors on IFN-γ transcription and Th1 cell differentiation in tilapia. The mRNA levels of T-bet, STAT1, and STAT4 were significantly higher in spleen lymphocytes activated by P+I (S4A Fig), PHA (Fig 5A), or CD3 plus CD28 mAbs (S4B Fig) than in control cells. Furthermore, the proportion of spleen lymphocytes in which STAT1 was phosphorylated markedly increased early after activation (Figs 5B and S4C). Moreover, overexpression of tilapia T-bet and STAT1 but not STAT4 elevated the transcription levels of IFN-γ in HEK 293T cells (Fig 5C), and the LUC activity driven by the IFN-γ promoter was enhanced by T-bet but not by STAT1 or STAT4 (Fig 5D), indicating that tilapia T-bet promotes IFN-γ expression by interacting with its promoter regions, whereas STAT1 is indirectly involved in this process. Additionally, STAT1 inhibition by the specific inhibitor Fludarabine [45] significantly reduced T-bet expression upon PHA- or CD3 mAb-induced T-cell activation (S4D Fig), suggesting that the T-bet-regulated IFN-γ transcription is STAT1 dependent. In this case, we knocked down STAT1 and T-bet to investigate whether the STAT1/T-bet axis is involved in Th1 cell differentiation in tilapia. Interference of STAT1 impaired the T-bet and IFN-γ expression induced by T-cell activation (Fig 5E), whereas T-bet interference compromised IFN-γ expression (Fig 5F); however, STAT1 or T-bet knockdown did not affect STAT4 expression (S4E and S4F Fig). Moreover, deficiency of STAT1 or T-bet diminished CD3^+^CD4^+^IFN-γ^+^ cells upon T-cell activation (Fig 5G). Notably, during *E*. *piscicida* infection, STAT1 or T-bet knockdown, which impaired the expression of key transcription factors and IFN-γ at the mRNA (S4G and S4H Fig) or protein level (Fig 5H), reduced the proportion (S4I and S4J Fig) and absolute number (Fig 5I–5L) of both CD3^+^CD4-1^+^ T cells and IFN-γ-producing CD3^+^CD4-1^+^ T cells. Thus, these findings highlight the central role of the STAT1/T-bet axis in regulating Th1 cell differentiation in tilapia. Not only gene knockdown but the blockade of STAT1 activity by Fludarabine also impaired the *E*. *piscicida-*induced IFN-γ expression and CD3^+^CD4^+^IFN-γ^+^ cells in spleen lymphocytes (Fig 5M and 5N), compromised bacterial clearance (S4K Fig), and eventually increased the mortality of tilapia (Fig 5O). Considering that IL-12 signaling and the transcription factor Runx3 are crucial for Th1 cell differentiation in mammals, we examined their expression during T-cell activation in tilapia. The mRNA expression of IL-12 receptors, but not Runx3, was induced upon PHA or TCR stimulation (S5 Fig), indicating that IL-12 signaling is associated with Th1 cell differentiation in tilapia as well. Together, our findings suggest that the conserved STAT1/T-bet axis is crucial for initiating Th1 cell differentiation in tilapia.

### IL-2 induces Th1 cell differentiation of tilapia via STAT5 and mTORC1 signaling

**Fig 6:**
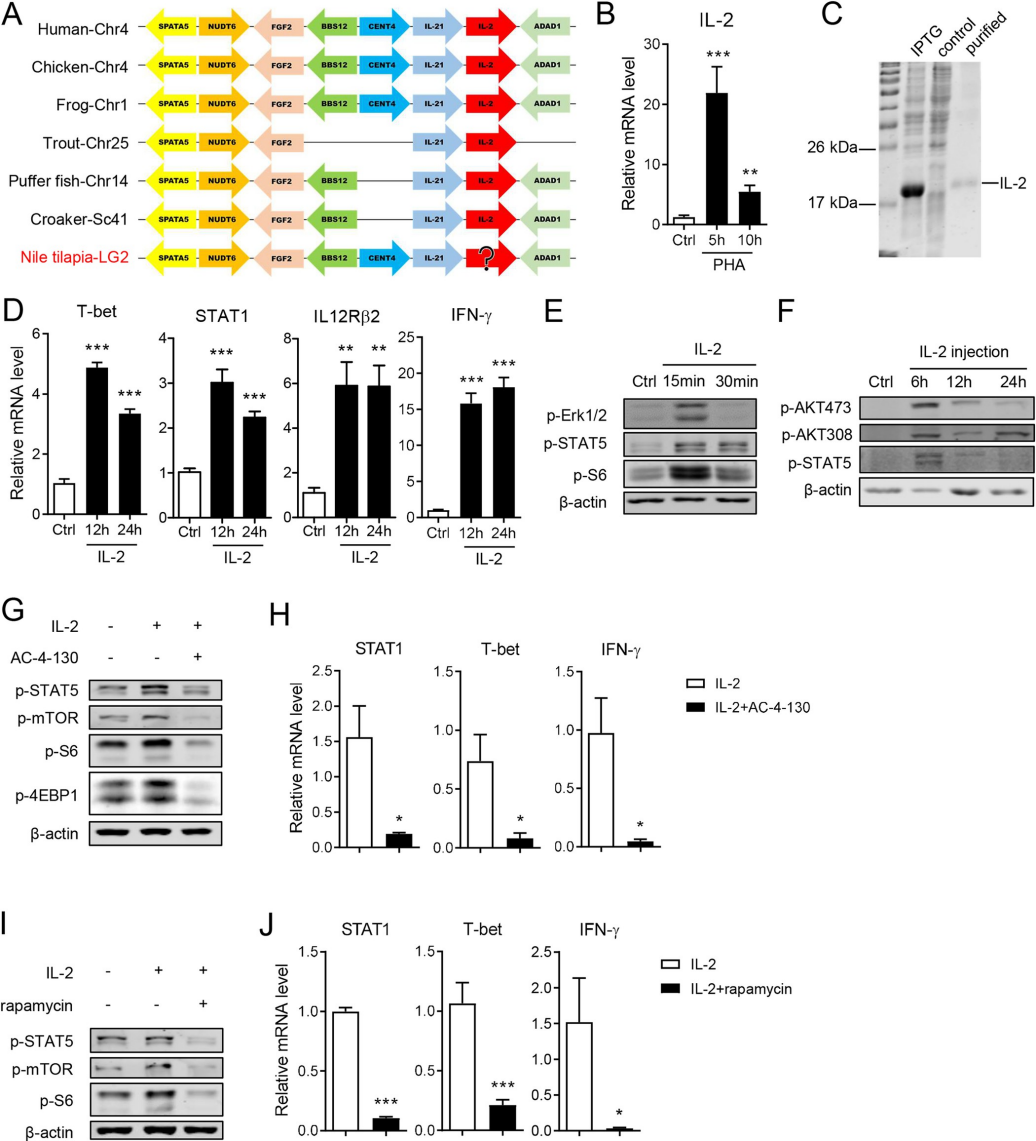
IL-2 promotes Th1 cell differentiation of tilapia by activating STAT5 and mTORC1 signaling. (**A**) Synteny analysis of tilapia IL-2 and other homology genes by comparative genomics. (**B**) Spleen leukocytes were stimulated with PHA, and mRNA levels of IL-2 were examined by qPCR, n = 6. (**C**) SDS-PAGE assay showed the recombination of tilapia IL-2 protein in *E*.*coli*. (**D, E**) Spleen leukocytes were treated with recombinant IL-2, and expression of indicated molecules were examined by qPCR (**D**, n = 6) or western blot (**E**) at indicated time points. (**F**) Tilapia individuals were *i*.*p*. injected with recombinant IL-2, the expression of indicated molecules were examined by western blot. (**G, H**) Spleen leukocytes were stimulated with recombinant IL-2 in the presence of STAT5 inhibitor AC-4-130 for 12 h. The expression levels of indicated molecules were examined by western blot (**G**) or qPCR (**H**, n = 4). (**I, J**) Tilapia was injected with mTORC1 inhibitor rapamycin for 2 days before spleen leukocytes were stimulated with recombinant IL-2 for 12 h. The expression levels of indicated molecules were examined by western blot (**I**) or qPCR (**J**, n = 6). These experiments were repeated for at least two independent times. *: *p*<0.05, **: *p*<0.01, ***: *p*<0.001, determined by a two-tailed Student’s t-test.

Differentiation of naïve CD4^+^ T cells into the Th1 lineage is tightly regulated by several cytokine signaling pathways. IL-2 initiates Th1 cell development by promoting the STAT5-dependent expression of IL-12Rβ2 and T-bet [46]. Although the IL-2 gene was not annotated in the Nile tilapia genome in NCBI GenBank, the genomic synteny analysis identified a gene located at the same locus as IL-2 homologues in other vertebrates (Fig 6A). The fact that this candidate gene exhibits a similar organizational structures, functional domains, tertiary structure, and phylogenic profile as the IL-2 homologues in other vertebrates (S6 Fig), suggests that this unannotated gene encodes tilapia IL-2. Upon PHA-induced T-cell activation, IL-2 expression in spleen lymphocytes was evidently upregulated (Fig 6B). Then, we developed an IL-2 recombinant protein to elucidate its potential role in triggering Th1 cell differentiation (Fig 6C). IL-2 stimulation elevated the transcription levels of T-bet, STAT1, and IL-12Rβ2 in spleen lymphocytes and eventually upregulated IFN-γ expression (Fig 6D). Mechanistically, the *in vitro* application or *in vivo* injection of IL-2 enhanced STAT5 phosphorylation (Fig 6E and 6F) and activated MAPK/ERK and mTOR pathways, as evidenced by the increased phosphorylation of ERK1/2 and AKT in spleen lymphocytes (Fig 6E and 6F). Because IL-2 initiated both STAT5 and mTORC1 signaling in tilapia, we further investigated the relationship between these pathways in regulating Th1 cell differentiation. Treatment of spleen lymphocytes with the STAT5 inhibitor AC-4-130 markedly decreased IL-2-induced STAT5 phosphorylation, which subsequently compromised the activation of mTORC1 signaling, as indicated by the diminished phosphorylation of mTOR, S6, and 4EBP1 (Fig 6G). Moreover, lymphocytes lacking STAT5 activity failed to elevate STAT1, T-bet, and IFN-γ upon IL-2 stimulation (Fig 6H). Remarkably, the blockade of mTORC1 signaling by rapamycin in turn reduced the IL-2-induced STAT5 phosphorylation (Fig 6I) and consequently impaired the expression of Th1 cell-related transcription factors and cytokines (Fig 6J). These observations indicate that downstream of IL-2 signaling, STAT5 and mTORC1 promotes Th1-cell differentiation via a bidirectional regulation and mutually reinforcing manner.

### mTORC1 ensures Th1 cell development in tilapia during *E*. *piscicida* infection

**Fig 7:**
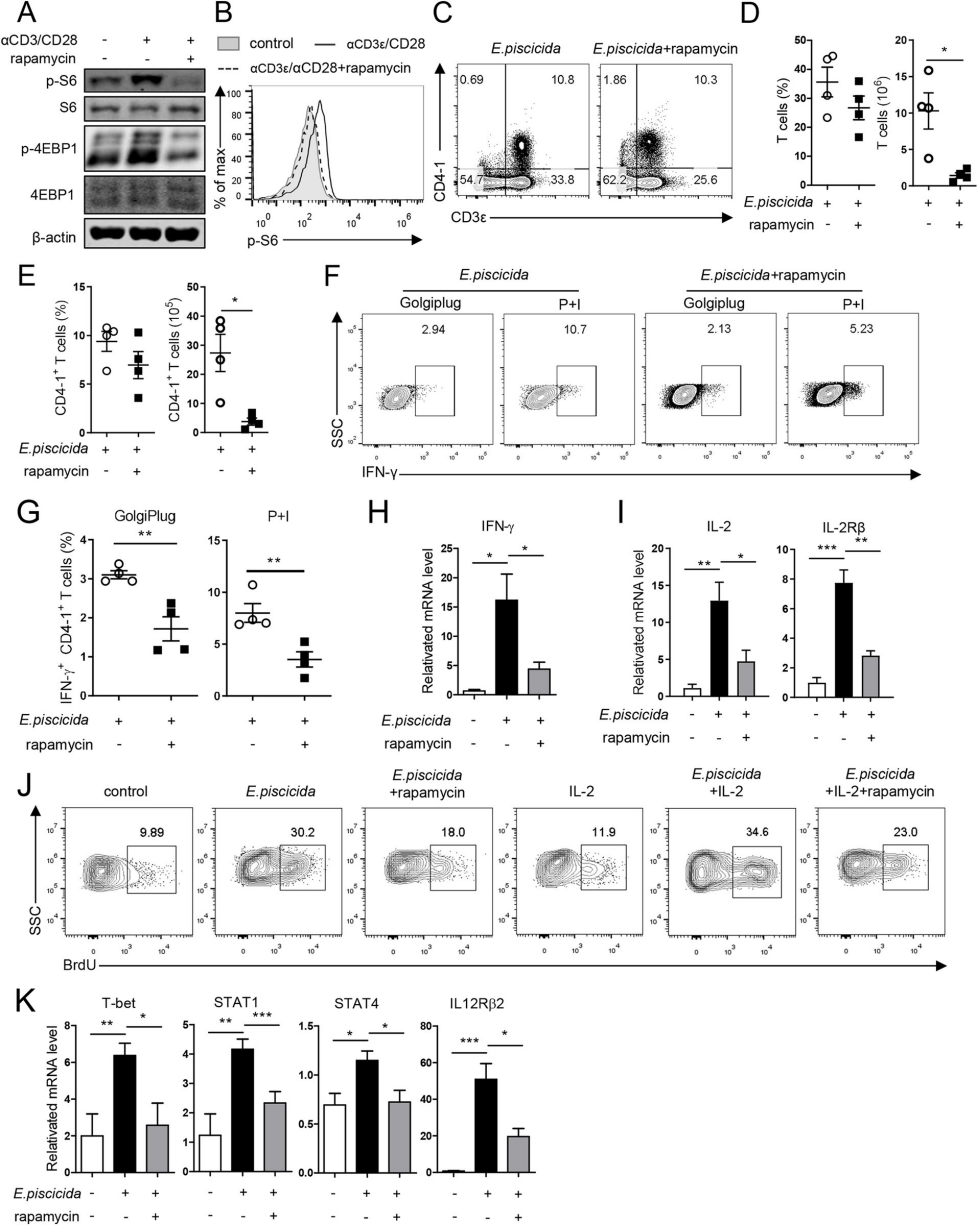
Tilapia requires mTORC1 signaling for Th1 cell development during *E*. *piscicida* infection. **(A, B)** Spleen leukocytes from rapamycin treated or untreated tilapia were stimulated with CD3ε/CD28 mAbs for 12 h. (**A**) Western blot assay showed protein or phosphorylation levels of S6 and 4EBP1 in spleen leukocytes. (**B**) Overlaid histograms showed the phosphorylation levels of S6 in CD4-1^+^ T cells. (**C-K**) Tilapia individuals that infected with *E*. *piscicida* were treated with rapamycin or PBS. (**C**) Flow cytometry showed the spleen leukocytes stained with CD4-1 and CD3ε mAbs on 7 DPI. (**D**) Scatter plot figures showed the percentage and absolute numbers of T cells, n = 4. (**E**) Scatter plot figures showed the percentage and absolute numbers of CD4-1^+^ T cells, n = 4. (**F, G**) Spleen leukocytes harvested from the tilapia on 7 DPI were stimulated with P+I or not with the presence of Golgiplug *in vitro* for 4 h, and flow cytometry (**F**) and Scatter plot figures (**G**) showed the percentages of IFN-γ^+^CD4+ T cells, n = 4. (**H, I, K**) The mRNA levels of indicated molecules were examined by qPCR on 4 DPI, n = 6. (**J**) Tilapia individuals that infected with *E*. *piscicida* were treated with rapamycin or PBS in the presence or absence of recombinant IL-2. Tilapia was *i*.*p*. injected with BrdU 1 day before sacrifice, and flow cytometry showed the BrdU staining on gated CD4-1^+^ T cells on 5 DPI. These experiments were repeated for at least two independent times. *: *p*<0.05, **: *p*<0.01, ***: *p*<0.001, determined by a two-tailed Student’s t-test.

The mTORC1 pathway helps naïve T cells to exit quiescence and regulates their differentiation into the Th1 lineage [47]. Our previous study revealing that mTORC1 plays a pivotal role in the T-cell response of tilapia [36], temps us to investigate whether and how this pathway contributes to Th1 cell development in fish. Spleen lymphocytes or CD3^+^CD4-1^+^ T cells exposed to TCR and costimulatory stimulation showed markedly higher S6 and 4E-BP1 phosphorylation (Fig 7A and 7B), indicating the potential involvement of mTORC1 signaling in the CD4^+^ T-cell response of tilapia. Then, the mTORC1 inhibitor rapamycin, which effectively blocked mTORC1 activity in activated T cells (Fig 7A) and CD4^+^ T cells (Fig 7B), was administered to tilapia during *E*. *piscicida* infection. Although mTORC1 inhibition did not affect the percentage of T-cell populations, it markedly reduced the absolute numbers of CD3^+^ T cells and CD3^+^CD4-1^+^ T cells compared with those in the untreated animals (Fig 7C–7E). Moreover, both the ability and capacity of CD3^+^CD4-1^+^ T cells to produce IFN-γ were markedly impaired in the absence of mTORC1 activity (Fig 7F and 7G). Consistent with the cellular evidence, the transcription level of IFN-γ in spleen lymphocytes was elevated upon *E*. *piscicida* infection, and this inducible expression was severely dampened by the rapamycin treatment (Fig 7H). Furthermore, lymphocytes lacking mTORC1 activity showed lower expression of IL-2 and IL-2R beta chain (Fig 7I), indicating an impairment of T-cell activation. Remarkably, mTORC1 deficiency hampered the proliferation of CD4-1^+^ T cells during *E*. *piscicida* infection, as indicated by the reduced BrdU incorporation in these cells (Fig 7J). Moreover, the addition of exogenous IL-2 slightly increased the proliferation of CD4-1^+^ T cells in both healthy and *E*. *piscicida*-infected tilapia, and this response was also found to be dependent on mTORC1 activity (Fig 7J). The induction of Th1 cell development by *E*. *piscicida* infection was supported by the upregulation of T-bet, STAT1, STAT4, and IL-12Rβ2 (Fig 7K), whereas mTORC1 deficiency markedly reduced the inducible expression of these crucial molecules (Fig 7K), suggesting that the transcriptional networks are regulated by the mTORC1 signaling pathway. Overall, our findings suggest that the mTORC1 signaling pathway regulates Th1 cell development in tilapia during *E*. *piscicida* infection by modulating cellular activation and proliferation and IFN-γ transcription.

### IFN-γ enhances Th1 cell polarization via STAT1/T-bet positive feedback

**Fig 8:**
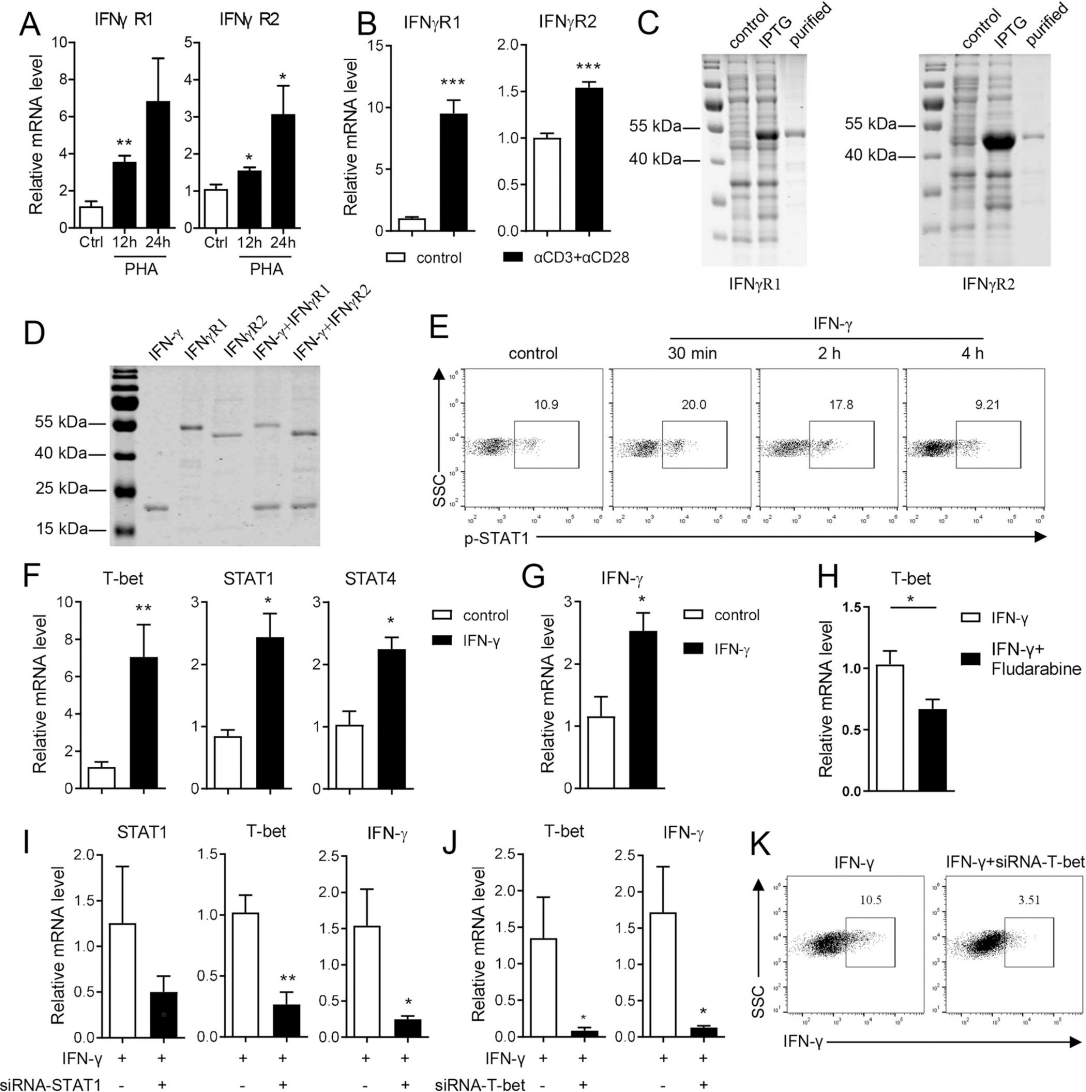
IFN-γ enhances tilapia Th1 cell commitment. (**A, B**) Spleen leukocytes were stimulated with PHA or CD3ε/CD28 mAbs for 12 h, and mRNA levels of IFNγR1 and IFNγR2 were examined by qPCR, n = 6. (**C**) SDS-PAGE assay showed the recombination of tilapia IFNγR1 and IFNγR2 with GST-tag in *E*.*coli*. (**D**) GST pull-down assay showed the interaction of tilapia IFN-γ with IFNγR1 and IFNγR2. (**E**) Flow cytometry showed the phosphorylation level of STAT1 in lymphocytes that stimulated with recombinant IFN-γ. (**F, G**) Spleen leukocytes were stimulated with recombinant IFN-γ for 12 h, and mRNA levels of indicated molecules were examined by qPCR, n = 5. (**H**) Tilapia was injected with STAT1 inhibitor for 2 days before spleen leukocytes were stimulated with recombinant IFN-γ for 12 h. The expression levels of T-bet were examined by qPCR, n = 6. (**I-K**) Spleen leukocytes from tilapia that *i*.*p*. injected with T-bet-specific, STAT1-specific or control siRNA for 2 days were harvested and stimulated with recombinant IFN-γ for 12 h. Relative mRNA levels of STAT1, T-bet and IFN-γ (**I, J**, n = 5), and the percentage of IFN-γ^+^ cells in gated CD3^+^CD4-1^+^ T cells (**K**) were examined. These experiments were repeated for at least two independent times. *: *p*<0.05, **: *p*<0.01, ***: *p*<0.001, determined by a two-tailed Student’s t-test.

Downstream of IFN-γ signaling, two IFN-γ receptors, IFNγR1 and IFNγR2, were identified in Nile tilapia. Both receptor chains were high similar to their respective mouse homologues in tertiary structure (S7A Fig), and they clustered well with their counterparts from other vertebrates (S7B Fig), suggesting their evolutionarily conserved properties. The transcription levels of IFNγR1 and IFNγR2 were markedly elevated upon induction of T-cell activation by PHA or anti-CD3 plus anti-CD28 mAbs (Fig 8A and 8B), indicating their potential participation in the T-cell response of tilapia. Recombinant GST fusion proteins encoding the extracellular domains of IFNγR1 and IFNγR2, respectively, were prepared (Fig 8C), and the GST pull-down assay using GST resins confirmed that both IFNγR1 and IFNγR2 interacted with tilapia IFN-γ *in vitro* (Fig 8D). Moreover, the addition of recombinant IFN-γ enhanced STAT1 phosphorylation (Fig 8E) and induced the expression of the transcription factors T-bet, STAT1, and STAT4 (Fig 8F), which eventually elevated the mRNA level of IFN-γ in spleen lymphocytes (Fig 8G). In addition, the blockade of STAT1 with Fludarabine impaired the IFN-γ-induced T-bet expression (Fig 8H), suggesting that IFN-γ promotes Th1 cell commitment through the STAT1/T-bet axis. This conclusion was further substantiated by the results of RNAi experiments, in which STAT1 or T-bet knockdown markedly reduced the IFN-γ-induced T-bet and/or IFN-γ expression (Fig 8I and 8J) and consequently decreased the proportion of IFN-γ-producing CD3^+^CD4-1^+^ T cells (Fig 8K). Therefore, our findings support the notion that Th1 cells in tilapia utilize a sophisticated STAT1/T-bet positive feedback loop to promote their commitment.

## Discussion

With the increasing environmental complexity and pathogen diversity, CD4^+^ T cells differentiate into a specific IFN-γ-secreting Th1 cell subset to resist intracellular pathogenic infections [1]. Elucidation of the emergence, function, and mechanism of specialized Th1 cells in early vertebrates would improve our understanding of the evolution of the adaptive immune system. Although recent studies have demonstrated that leukocytes, CD4-1^+^ lymphocytes, or CD3^+^ T cells of bony fishes express IFN-γ at the transcriptional level [27,32,48–50], indicating that Th1-like cells have emerged in these fish species, the formal existence of CD3^+^CD4-1^+^IFN-γ^+^ T cells, their detailed function, and differentiation mechanism have not been well elucidated. In the present study, we identified a population of CD3^+^CD4-1^+^IFN-γ^+^ Th1 cells in Nile tilapia, and investigated their immunological function in resisting intracellular bacterial infection and the mechanism underpinning their differentiation.

Although a few bony fish species are reported to have lost the CD4/MHCII axis [24,25], most species encode CD4 receptors. However, the number of CD4 molecules differs among bony fish species due to genome or gene duplication events during evolution. For example, trout, catfish, ginbuna crucian carp, Japanese flounder, and tilapia have two CD4 molecules, CD4-1 and CD4-2 [51–54]; zebrafish and Atlantic salmon are reported to encode three, CD4-1, CD4-2a and CD4-2b [32,55]; and some bony fish such as fugu, sea bass, carp, and Atlantic halibut have only one CD4 molecule [54,56–58]. With regard to the Th1 subset, leukocytes or CD4-1^+^ lymphocytes from many bony fish species such as zebrafish, rainbow trout, grass carp, tilapia, and mandarin fish have been reported to express IFN-γ at the mRNA level, and its transcription is markedly induced by pathogenic infection or PAMP stimulation [27,32,37,50,59]. However, to date, the existence of a CD3^+^CD4-1^+^IFN-γ^+^ T-cell population representing Th1 cells has not been reported in bony fish. In the present study, we demonstrated that the bony fish Nile tilapia possess a population of CD3^+^CD4-1^+^ T cells and that a proportion of these cells secrete IFN-γ protein during T-cell activation or bacterial infection. Notably, by using approaches such as CD4-1^+^ T-cell depletion and IFN-γ blocking, we showed that tilapia Th1 cells play a crucial role in resisting intracellular pathogen infection by producing IFN-γ and enhancing macrophage activity. Our findings thus support the notion that, before the emergence of tetrapods, CD3^+^CD4-1^+^IFN-γ^+^ Th1 cells already existed in some bony fish and that the reliance on Th1 cells to fight intracellular pathogenic infection represents an evolutionarily ancient strategy. Nevertheless, the present evidence is not sufficient to answer whether the emergence of Th1 cells in bony fish represents a complete innovation of the adaptive immune system or simply a refinement of an evolutionarily ancient IFN-γ effector program. In the jawless vertebrate lamprey, which possesses variable lymphocyte receptor (VLR)-based T-like cells and a CD4-like receptor [60,61], IFN-γ is virtually absent. In contrast, cartilaginous fish have evolved TCR-expressing CD8^+^ T cells and encode the IFN-γ gene, but the existence of Th cells is still questioned owing to the lack of a CD4 co-receptor and several cytokines, cytokine receptors, and transcription factors [13,62]. Therefore, how IFN-γ and transcription factors have been rewired at the molecular level and in CD4^+^ T cells to determine the emergence of Th1 cells remains an immunological question that needs further elucidation.

Essentially, the direct determinants of Th1 cell differentiation are transcriptional networks downstream of cytokine signaling. Mammalian CD4^+^ T cells utilize a set of cytokine-controlled STATs and T-bet to regulate and maintain Th1 cell development. For example, IL-2 promotes T-cell proliferation and Th1 cell differentiation by inducing STAT5 phosphorylation and STAT1 and T-bet expression [46], whereas IL-27 and IL-12 enhance T-bet expression through STAT1 and STAT4, and play a critical role in the early and late stages of Th1 cell differentiation, respectively [63,64]. These configurations not only provide the microenvironment and immune signaling for CD4^+^ T cells, but also determine the direction of Th cell differentiation and the nature of the immune response [1]. Recent evidence suggests the existence of the configurations necessary for Th1 cell differentiation in various bony fish species, including the cytokines IL-2, IL-27, and IL-12 [65–67] and transcription factors T-bet, STAT1, and STAT4 [68–70], and it is reported that their expression is markedly induced during bacterial or viral infection. Notably, IL-2 signaling couples the MAPK and mTORC1 axes to promote IFN-γ mRNA expression in leukocytes of large yellow croaker, whereas IL-2, IFN-γ, or IL-6 treatment increases the percentage of T-bet^+^ T cells in Japanese flounder [71,72], indicating that these cytokines potentially regulate the differentiation of Th1 cells in bony fish. In the present study, we showed that overexpression of tilapia STAT1 or T-bet elevated IFN-γ expression and that T-bet but not STAT1 enhanced the activity of IFN-γ promoter. Furthermore, the blockade or knockdown of STAT1 impaired T-bet and IFN-γ expression, compromised the development of CD3^+^CD4-1^+^IFN-γ^+^ Th1 cells, and weakened the anti-bacterial immune response of tilapia. These findings thus established that a STAT1–T-bet axis regulates the differentiation of Th1 cells in tilapia. Upstream of this signaling axis, IL-2 induced the expression of STAT1 and T-bet in tilapia lymphocytes and further promoted IFN-γ transcription, likely via STAT5 and mTORC1 signaling. Because IL-2 has also been reported to upregulate IFN-γ and T-bet expression or increase the T-bet^+^ T cells in other bony fish [71,72], it is speculated that these early vertebrates use a similar IL-2 signaling pathway as that in mammals to facilitate Th1 cell differentiation. Notably, we further revealed that tilapia IFN-γ bound its receptors IFNγR1 and IFNγR2 to promote STAT1-dependent T-bet expression, which in turn enhanced Th1 cell commitment, thus forming a positive feedback regulatory loop. Although our attempts to induce the differentiation of tilapia CD3^+^CD4-1^+^IFN-γ^+^ T cells *in vitro* were unsuccessful, we have established the regulatory mechanism underpinning Th1 cell differentiation in this fish species, and we speculate that the strategies determining the fate of Th1 cells have been programmed early during vertebrate evolution.

As the core integrator of signal transduction, mTORC1 pathway responds to multiple cues in the microenvironment, such as nutrients, growth factors, and immune signaling, thus playing an essential role in regulating T-cell differentiation and function [47]. Th1 cell development depends significantly on the mTORC1 pathway; a lack of mTORC1 activity in T cells caused by mTOR or Rheb knockout markedly impairs Th1 cell differentiation in mice [12,73]. mTORC1 determines Th1 cell differentiation mainly by regulating the activity of STAT, which further promotes the expression of the transcription factor T-bet [73]. In addition, knockdown of SOCS3 in Rheb-deficient T cells restores Th1 cell differentiation, suggesting that mTORC1 also controls Th1 lineage development by suppressing SOCS3 [73]. The observations that mTORC1 activity is essential for fish lymphocytes to express IFN-γ in our previous study and another study [36,72] prompted us to investigate the potential role of this pathway in Th1 cell development in tilapia. Notably, we found that mTORC1 regulates the development of CD3^+^CD4-1^+^IFN-γ^+^ T cells in tilapia during intracellular bacterial infection at least by promoting the proliferation of CD3^+^CD4^+^ T cells and expression of the transcription factors STAT1 and T-bet. Moreover, we found that mTORC1 activity, which is involved in the differentiation of tilapia Th1 cells, was driven by IL-2. Downstream of IL-2 signaling, mTORC1 closely communicated with STAT5 to regulate Th1 cell development in a mutually reinforcing manner. Therefore, we speculate that mTORC1-controlled Th1 cell differentiation is an ancient and sophisticated program, which ensured that early vertebrates could optimally respond to the increasing environmental complexity and pathogen diversity during evolution.

The critical roles of adaptive immune system in bony fish have been neglected to some extent, and these animals were generally believed to rely more on innate immunity to resist pathogenic infections. Our recent studies have demonstrated that tilapia possess well-evolved T-cell immunity and sophisticated regulatory mechanisms. For example, mTORC1 couples immune signaling with metabolic reprograming, NF-κB couples TCR and IL-17 signals, Ca^2+^–calcineurin axis triggers NFAT nuclear translocation, and the MAPK/ERK cascade initiates c-Myc-mediated glycolysis to ensure proper activation, proliferation, and anti-infection immune response of tilapia T cells [33–36]. On the other hand, it was reported that rainbow trout employs IgM^+^ and IgT^+^ B cells and corresponding antibodies to perform their respective functions in systemic and mucosal humoral immunity [74,75]. These findings indicate that at least some bony fish possess a well-evolved adaptive immune system. Remarkably, in the present study, we found that the CD4^+^ T cells of tilapia can differentiate into a Th1 cell subset, thus contributing to a more specific immune response. In mammals, Th1 cells produce large amounts of IFN-γ, which enhances macrophage activity, and exert bactericidal effects by releasing NO and ROS [4]. Moreover, IFN-γ collaborates with LT-α to recruit and activate inflammatory cells to resist infection [4]. In addition, IL-2 produced by Th1 cells promotes the differentiation of CD8^+^ effector T cells for the cytotoxicity function [76]. Here, in tilapia, we found that intracellular bacterial infection triggers the proliferation of CD4-1^+^ T cells and the development of the CD3^+^CD4-1^+^IFN-γ^+^ Th1 subset; IFN-γ and Th1 cells facilitated infection elimination and considerably improved survival. These results demonstrate that Nile tilapia has evolved a specialized Th1 cell subset to resist intracellular pathogen infection. The proportion of CD3^+^CD4-1^+^IFN-γ^+^ Th1 cells in tilapia is lower than that in mammals [77], which may be attributed to the differences between fish and mammals regarding their living environment, cold- versus warm-bloodedness, and other physiological characteristics. In fact, the blockade or knockdown of signaling molecules in the present study, using specific inhibitors or siRNA, cannot exclude effects from other cell lineages. In future studies, knockout models of tilapia driven by LCK or CD4-Cre are expected to verify the intrinsic regulatory roles of mTORC1, STAT1, or T-bet in Th1 cell differentiation. Nevertheless, our findings have demonstrated that tilapia has a set of anti-infection mechanisms ranging from T-cell activation and proliferation to Th1 cell differentiation. Admittedly, the knowledge regarding adaptive immunity in bony fish lags far behind that in mammals, but this gap will continue to narrow as the defense strategies of fish are further investigated.

**Fig 9:**
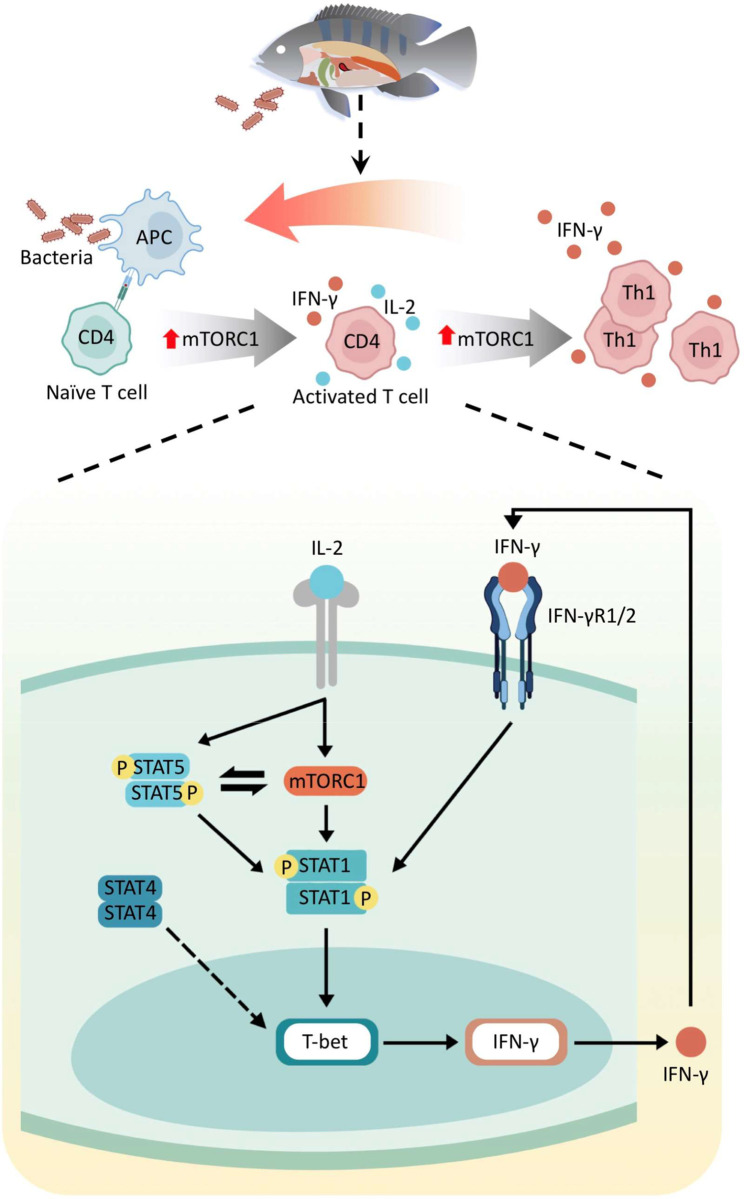
IL-2-mTORC1 signaling coordinates STAT1/T-bet axis to ensure Th1 cell differentiation and anti-bacterial immune response in tilapia.

In summary, the present findings demonstrate that Nile tilapia possess specialized CD3^+^CD4-1^+^IFN-γ^+^ Th1 cells to resist intracellular bacterial infection, and they employ a set of sophisticated mechanisms to ensure the proper development of this cell lineage. Upon infection, the TCR signal triggers the mTORC1 pathway that promotes CD4-1^+^ T-cell activation and proliferation (Fig 9). Activated T cells produce IL-2, which further enhances the STAT5 and mTORC1 signaling and initiates the STAT1/T-bet axis-regulated IFN-γ transcription and CD3^+^CD4-1^+^IFN-γ^+^ T cell development (Fig 9). Moreover, IFN-γ binds to its receptors and promotes Th1 cell polarization through a STAT1/T-bet axis-mediated positive feedback loop and facilitates the anti-bacterial immune responses by enhancing macrophage activity (Fig 9). These findings provide valuable evidence and novel perspectives for understanding the evolution of adaptive immunity.

## Materials and methods

### Ethics statement and animals

Nile tilapia larvae were purchased from an aquatic farm in Guangzhou, Guangdong Province, China, and maintained in a circulating water system with continuous aeration at 28°C until they reached to 10 cm, at the Biological Station of East China Normal University. Fish were fed twice one day with commercial pellets, and were randomly transferred to independent tanks before experiment. BALB/c mice were purchased and seeded in Minhang Laboratory Animal Center of East China Normal University. All animal care and experimental procedures were performed in accordance with the Guide for the Care and Use of Laboratory Animals of the Ministry of Science and Technology of China and were approved by the East China Normal University Experimental Animal Ethics Committee, with an approval number m20190108. All efforts were made to minimize the pain of animals.

### Cell lines

HEK293T, BOSC23 and NIH/3T3 cells were purchased from the American Type Culture Collection (ATCC, Manassas, VA, USA). The mouse myeloma SP2/0 was kindly gifted by the Ocean University of Shanghai, China. HEK 293T, BOSC23, and NIH/3T3 cells were cultured at 37°C, 5% CO_2_ in DMEM medium supplemented with 10% FBS and 1% penicillin/streptomycin. The SP2/0 cells were cultured at 37°C, 5% CO_2_ in GIT medium.

### Sequence, structure and phylogenic analysis

The cDNA and amino acid sequences of related genes were obtained from National Center for biotechnology information (NCBI). Chromosomal locations of IFN-γ genes from human, chicken, turtle, frog, zebrafish, and Nile tilapia were obtained from NCBI, and gene structure information of IFN-γ from other species was downloaded from NCBI. Multiple sequence alignments were generated by Clustalx and presented by The Sequence Manipulation Suite (http://www.bio-soft.net/sms/index.html). Phylogenetic tree was constructed in MEGA7 using neighbor-joining (NJ) method with 1000 bootstrap replications. The conserved domains were predicted using simple modular architecture research tool (http://smart.embl-heidelberg.de/), and the domain organization was displayed by DOG 2.0 software. The protein tertiary structure was predicted by SWISS-MODEL (https://swissmodel.expasy.org/interactive) and displayed by PyMOL software. Accession numbers for these sequences used were listed in S1 Table.

### Recombinant protein preparation

The fragments encoding Nile tilapia IFN-γ and IL-2 (exclude signal peptide) were ligated into vector pET-28a (+), and fragments encoding the extracellular domains of IFNγR1 and IFNγR2 were ligated into vector pGEX-4T-3. The recombinant plasmids were transformed into transetta (DE3) chemically competent cells, and then induced by 0.5 mM IPTG at 37°C for 4 h. Recombinant protein IFN-γ and IL-2 with His-tag were purified by affinity chromatography using Ni-NTA resins. Subsequently, purified protein was re-folded by gradient dialysis at 4°C, and finally dialyzed in PBS thoroughly. Recombinant protein of IFNγR1 and IFNγR2 with GST-tag were purified by affinity chromatography using GST resins. The obtained proteins were concentrated with a microcon-10 kDa molecular mass centrifugal filters unit (Millipore), measured concentration with BCA Protein Assay Kit (Sangon Biotech), and stored at -80°C before use. Accession numbers for the sequences used were listed in S1 Table.

### Retrovirus packaging and cell infection

The full length of coding cDNA sequence for Nile tilapia CD4-1 was cloned into MIGR1 plasmid. Subsequently, MIGR1-CD4-1 plasmid and helper plasmid were co-transfected into BOSC23 cells to produce retrovirus. The BOSC23 cells were seeded in 6 cm dish at 2×10^6^ cells overnight. After the medium was replaced by 3 mL fresh DMEM with 1‰ chloroquine, the cells were then co-transfected with 10 μg MIGR1-CD4-1 plasmid and 5 μg helper plasmid in the presence of 50 μL 2.5 M of CaCl_2_ and 500 μL 2×HEBS. Eight hours after transfection, the culture medium was replaced by fresh DMEM, and the cell supernatant containing retrovirus were collected at 48 h after transfection. NIH/3T3 cells were seeded in 6 cm dish at 2×10^5^ cells overnight, and 120 μL of retrovirus was used to infect NIH/3T3 cells in the presence of 5 μg/mL polybrene. At 4 h after infection, 500 μL fresh DMEM medium was added to dilute the virus, and the cell medium was replaced by 4 mL fresh DMEM medium on the other day. The infected cells were collected at 48 h after infection, and were used for flow cytometry and mouse immunization.

### Monoclonal antibodies (mAbs) preparation

Anti-tilapia CD3 and CD28 mAbs were previously developed by our lab. For IFN-γ mAb preparation, 100 μg purified IFN-γ protein was thoroughly emulsified with Freund’s complete adjuvant and immunized BALB/c mice by intraperitoneal (*i*.*p*.) injection. Two weeks later, another immunization with the same dose of antigen was *i*.*p*. injected using Freund’s incomplete adjuvant. For the last two immunizations, mice were immunized by intravenous injection of IFN-γ protein in PBS at 1-week intervals. To develop CD4-1 mAb, the NIH/3T3 cells expressing Nile tilapia CD4-1 were used to immunize BALB/c mice by *i*.*p*. injection for four times with the same intervals as above. Then, BALB/c mice were euthanized and splenocytes were fused with SP2/0 cells in the presence of polyethylene glycol (PEG). The fused cells were seeded in 96-well plates together with mouse thymocytes. After growing in the medium containing hypoxanthine, aminopterin and thymidine (HAT) over 10 days, the supernatants of hybridomas were screened for positive clones by ELISA and flow cytometry. The positive hybridomas were sub-cloned by limiting dilution method. The positive hybridomas were then *i*.*p*. injected into BALB/c mice to induce ascites, and mAbs were purified from ascites using protein G agarose (Invitrogen) and labelled with FITC or biotin.

### ELISA assay

ELISA plate was coated with 5 μg recombinant IFN-γ at 4°C overnight. After blocked with PBS containing 1% BSA at 37°C for 1 h, 100 μL of hybridomas supernatant was added and incubated at 37°C for another 1 h. After washed with PBST for 3 times, the samples were stained with goat anti-mouse IgG AP-conjugate secondary antibody at 37°C for 1 h, followed by incubating with 0.1% (w/v) p-nitrophenyl phosphate (pNPP) for 30 min. Finally, the reaction was stopped by 2 M NaOH, and then the values of OD (405 nm) were measured with microplate reader.

### Leukocyte isolation

The leukocytes were isolated from Nile tilapia spleen and head kidney according to our previous description [36]. Briefly, the spleen and head kidney were grinded in pre-cooled Leibovitz’s L-15 medium (Gibco), and filtered through nylon mesh. The cell suspension was added slowly onto a 52%/34% discontinuous density-gradient percoll (GE Healthcare). After centrifuged at 500 g, room temperature for 35 min with the lowest acceleration and deceleration, the cells between 34% and 52% percoll were collected for indicated assay.

### Immunofluorescence assay

The spleen leukocytes were spun onto slide by Cytospin (Thermo) and fixed with methanol at room temperature for 5 min, and then blocked with PBS containing 1% BSA at 37°C for 1 h. After 3 times’ wash with PBST, the cells were incubated with mouse anti-tilapia CD4-1 mAb at 37°C for 1 h, and stained with PE-conjugated goat anti-mouse IgG secondary antibody for another 1 h. The samples were then washed, and followed by the staining with FITC-conjugated mouse anti-tilapia CD3ε mAb. Finally, the cells were mounted with antifade mounting medium containing Hoechst 33342. The images were acquired by fluorescence microscope and analyzed by photoshop CS6.

### Leukocyte stimulation

The tilapia leukocytes that cultured at 28°C, 5% CO_2_, in DMEM containing 10% FBS and 1% penicillin/streptomycin were stimulated with phorbol 12-myrustate 13-acetatae (PMA, 50 ng/mL) plus ionomycin (500 ng/mL) or 2 μg/mL of PHA or 2 μg/mL α-CD3ε mAb at 28°C for indicated time. Then, the cells were harvested for qPCR, flow cytometry and western blot assay. For α-CD3ε/α-CD28 stimulation, spleen leukocytes were activated with plate-bound α-CD3ε (2 μg/mL) plus soluble α-CD28 (2 μg/mL) at 28°C for indicated time, and the activated cells were harvested for assays.

### Flow cytometry and cell sorting

To identify the CD3^+^ and CD4-1^+^ T-cell population, tilapia leukocytes were collected and incubated with CD4-1 mAb, followed by staining with PE-goat anti-mouse IgG secondary antibody. Subsequently, the cells were stained with FITC-conjugated CD3ε mAb and live/dead-Violet. Incubations of primary and secondary antibodies are on ice for 30 min, and two times of wash with FACS buffer (PBS with 2% FBS) were performed after each incubation. To identify IFN-γ producing cells, the leukocytes were stimulated with 50 ng/mL PMA plus 500 ng/mL ionomycin in the presence of 1 μg/mL of Golgiplug at 28°C for 4 h. After cell surface staining as above, cells were fixed with BD cytofix/cytoperm buffer on ice for 30 min, and washed twice with 1× BD perm/wash buffer. Subsequently, cells were stained with biotin-conjugated mouse anti-tilapia IFN-γ, and followed by APC-Streptavidin staining. For p-STAT1 staining, leukocytes were fixed with Foxp3 Fixation/Permeabilization working solution (eBioscience) on ice for 2 h, and then stained with Alexa Fluor 647 conjugated-p-STAT1 (Tyr701) Rabbit mAb (CST) on ice for 30 min. Stained cells were then washed twice with 1× permeabilization buffer. All the samples were analyzed by BD LSRFortessa flow cytometer, and data were analyzed using FlowJo software. For cell sorting, head kidney macrophages were gated according to our previous study [36], and head kidney leukocytes were stained with CD3 and CD4-1 as above. CD3^+^CD4-1^+^ T cells and macrophages were sorted using a BD FACSAria II flow cytometer.

### Western blot analysis

The cells were lysed in NP40 lysis buffer containing 1mM PMSF, 1‰ protease inhibitor and 1% phosphorylase inhibitor on ice for 30 min. After centrifuged, the supernatant was acquired and separated by 12% SDS-PAGE, and then transferred to nitrocellulose membrane. The nitrocellulose membrane was blocked with 4% skim milk powder in PBST for 1 h, and incubated with anti-tilapia IFN-γ mAb or 1:1000 diluted primary antibody against S6, 4EBP1, phospho-S6 (Ser240/244), phospho-4EBP1 (Thr37/46), phosphor-STAT5 (Tyr694), phosphor-Erk1/2 (Thr202/Tyr204), phosphor-Akt (Thr308), phosphor-Akt (Ser473), and β-actin from Cell Signaling Technology at 4°C overnight, and further incubated with goat anti-rabbit or mouse IgG H&L 800 or 680 (Abcam). To detect the overexpressed IFN-γ in 293T cells, the nitrocellulose membrane was incubated with anti-tilapia IFN-γ mAb, followed by Ap-conjugated goat anti-mouse IgG secondary antibody (Southern Biotech), and visualized with freshly prepared substrate solution (100 mM NaCl, 100 mM Tris, and 5 mM MgCl_2_; pH 9.5) containing nitroblue tetrazolium (Sigma) and 5-bromo-4-chloro-3-indolylphosphate (Sigma). Images were acquired by Odyssey CLx Image Studio.

### *Edwardsiella piscicida* infection

*Edwardsiella piscicida* was kindly gifted by the East China University of Science and Technology, and was cultured in Tryptone Yeast extract Broth (TYB) at 37°C overnight. After centrifuged at 6000 g for 3 min, the bacteria were collected and washed with PBS, and then resuspended in PBS to the OD_600_ value of 1.0. Nile tilapia were *i*.*p*. injected of 100 μL *E*. *piscicida* on the concentration of 2×10^7^ CFU/mL. The control group were injected with the same volume of PBS. Spleen leukocytes were isolated for assays on the indicated days.

### Depletion of CD4-1^+^ cells

Depletion of CD4-1 cells were performed according to a previous report with modification [78]. Tilapia individuals were *i*.*p*. injected with 20 μg of mouse anti-tilapia CD4-1 or mouse IgG1 (Catalog #401402, BioLegend) as isotype control antibody. After 24 h, the tilapia was *i*.*p*. injected with 50 μL tilapia anti-mouse IgG1 serum or tilapia control serum, respectively. Nine days after mAbs injection, spleen leukocytes were obtained, and the CD3ε^+^CD4-1^+^ T cells were stained and examined by flow cytometry. The nondepleted and CD4-1-depleted fish were then infected with *E*. *piscicida*, and animals were sacrificed on indicated days for assay.

### Inhibitor treatment

STAT1 inhibitor Fludarabine, STAT5 inhibitor Ac-4-130, mTORC1 inhibitor rapamycin and Brefeldin A were purchased from MedChemExpress. Nile tilapia was *i*.*p*. injected with 5 mg/kg Fludarabine or 1 mg/kg rapamycin every two days during *E*. *piscicida* infection. Nile tilapia was *i*.*p*. injected with 13 mg/kg Brefeldin A to block cytokine secreting at 6 h before sacrifice, and the spleen leukocytes were isolated for IFN-γ producing by flow cytometry or western blot on day 5 or 8 post infection. For the STAT5 inhibition, the isolated spleen leukocytes were treated with 5 mM Ac-4-130, and the cell were stimulated with or without recombinant IL-2 for 12 h.

### siRNA interference

Three pairs of tilapia T-bet or STAT1 specific siRNA, and one pair of negative control siRNA were designed by and ordered from GenePharma company. The primer sequences were listed in S2 Table. Tilapia were *i*.*p*. injected with 3 μg specific siRNA or control siRNA in 100 μl 1:500 diluted Lipo6000. Two days later, the spleen leukocytes were isolated and stimulated with 2 μg/ml plate-bound α-CD3ε for 12 h, and the activated cells were harvested for assays. For *in vivo* experiments, tilapia infected with *E*. *piscicida* were *i*.*p*. injected with 3 μg T-bet specific siRNA or STAT1 specific siRNA on day 2 and 4 post infection. The spleen leukocytes were isolated on indicated days for assays.

### Cytokines treatment

For *in vitro* treatment, the spleen leukocytes isolated from Nile tilapia were cultured in DMEM (10% FBS) and stimulated with 2 μg/ml recombinant IFN-γ or IL-2 for indicated times. Stimulated cells were collected for quantitative RT-PCR (qPCR) analysis, flow cytometry and western blot. For IFN-γ *in vivo* treatment, Nile tilapia were *i*.*p*. injected with 1 mg/kg recombinant IFN-γ each day during *E*. *piscicida* infection. At 5 days after infection, the bacterial titer in spleen was examined by coating the TYB solid medium and counting the number of colonies. For IL-2 *in vivo* treatment, Nile tilapia were *i*.*p*. injected with 1 mg/kg recombinant IL-2 for indicated time, and spleen leukocytes were isolated for western blot assay.

### Transfection and Dual-luciferase assay

Nile tilapia IFN-γ promoter (from -2000 to +0 bp) was cloned and ligated into pGL3 vector. The coding regions of T-bet, STAT1 and STAT4 were cloned into pcDNA3.1 vector. For dual-luciferase assay, 10^4^ of HEK 293T cells were seeded in 24-well plates overnight, and co-transfected with TF expression plasmid (pcDNA3.1-T-bet, STAT1 or STAT4), reporter genes (pGL3-IFN-γ promoter), and pRL-TK plasmid in 100 μL opti-DMEM medium containing 1 μL lipofectamine 2000. Six hours after transfection, the medium was replaced by fresh DMEM, and the cells were collected at 48 h after transfection for assay. The Luc activities were measured according to the manual of Dual-Luciferase Reporter Assay System (Promega). Cells were harvested and lysed by 1×PLB, and Luc activities were measured using the luminometer after mixing LAR II, and mixed Stop & Glo Reagent, respectively.

### Pull-down assay

The Recombinant protein GST tag-IFNγR1 and GST tag-IFNγR2 were incubated with anti-GST-Agarose beads at 4°C overnight, respectively. The beads were then washed 4 times with resin buffer, and then incubated with the recombinant His-tag IFN-γ at 4°C for 4 h. The beads were precipitated, washed, and resuspended in 5×SDS sample buffer, then boiled and separated by 12% SDS-PAGE, and stained with Coomassie brilliant blue. Images were acquired by Odyssey CLx Image Studio.

### Quantitative RT-PCR (qPCR)

Total RNA was extracted with TRIzol reagent (Invitrogen) according to the manufacturer’s instructions. After treated with gDNA pure to remove genomic DNA, the total RNA was used as template to synthesize the first strand of cDNA using 2×NovoScript Plus 1st Strand cDNA Synthesis SuperMix (Novoprotein). Then, the qPCR was performed with NovoStart SYBR qPCR SuperMix Plus (Novoprotein) on a QuantStudio 5 Real-Time PCR Instrument (Applied Biosystems). β-actin was used as internal reference, and the expression of target genes were analyzed by 2^-△△Ct^ method. The gene information and primer sequences were listed in S2 Table.

### BrdU incorporation

Nile tilapia was *i*.*p*. injected with 0.75 mg BrdU (Sigma) in 200 μL PBS, at 16 hours before the spleen leukocytes were isolated for assay. After the cells were stained with surface CD3ε and CD4-1 as above, they were fixed with BD Cytofix/Cytoperm Buffer on ice for 30 min, and then washed twice with BD Perm/Wash Buffer. After that, the cells were treated with BD Cytoperm Plus Buffer for 10 min and BD Cytofix/Cytoperm Buffer for 5 min on ice, respectively, and then digested with 300 μg/mL DNase at 37°C for 1 h. Samples were subsequently stained with FITC-anti-BrdU antibody (BD) at room temperature for 20 min and analyzed with flow cytometry.

### Phagocytosis assay

FITC-labelled *E*. *piscicida* was incubated with leukocyte suspension, sorted macrophages, or sorted macrophage plus sorted CD4-1^+^ T cells containing 5 μg of recombinant IFN-γ or not at 28°C for 6 or 12 h. After that, 0.2% trypan blue was added to quench the fluorescence from attached bacteria. The macrophage population of Nile tilapia was gated by flow cytometry according to our previous study [36], and their phagocytosis ability was then examined.

### IFN-γ mAb blocking

The head kidney leukocytes, or sorted head kidney macrophage plus CD4-1^+^ T cells were activated with 2 μg/mL plate-bound α-CD3ε, and 10 μg/ml IFN-γ mAb was added to block the IFN-γ. FITC-labelled *E*. *piscicida* were then added into the cells and incubated for 12 h. The macrophage population of Nile tilapia was gated and to measure their phagocytosis ability.

### Statistical analysis

Prism version 8.0 software (GraphPad Software Inc., San Diego, CA, USA) was used for statistical analysis. The statistical significance of results was analyzed using two-tailed, unpaired, Student’s t-test. Animal survival data were analyzed by log-rank analysis. The significance was indicated by *, *p* < 0.05, **, *p* < 0.01, ***, *p* < 0.001.

## Supporting information

S1 Fig Evolutionary properties of the Nile tilapia IFN-γ. (**A**) The synteny analysis of tilapia IFN-γ and its homologues from other vertebrates. (**B**) Gene structures of tilapia IFN-γ and its vertebrate counterparts. (**C**) Multiple sequence alignment of IFN-γ from tilapia and other vertebrates. Amino acid residues with 60% identity are in black, and similar residues are in dark gray. Some important regions were marked out, with underline for signal peptide, with overline for NLS. The sequence of signature motif ([I/V]-Q-X-[K/Q]-A-X2-E-[L/F]-X2-[I/V]) was boxed. (**D**) Phylogenetic tree constructed with the amino acid sequences of IFN-γ from the indicated species. Phylogenetic tree was constructed in MEGA7 by using neighbor-joining (NJ) method with 1000 bootstrap replications. The accession numbers of selected sequences were listed in S1 Table.
(PDF)

S2 Fig Analysis of head kidney CD4-1^+^ T cells in the CD4-1 depleted tilapia. CD4-1^+^ cells were depleted as described in Fig 3F. **(A)** Flow cytometry showed the percentage of CD3^+^CD4-1^+^ T cells in head kidney lymphocytes of CD4-1-depleted or non-depleted tilapia on 9-day post depletion. **(B)** Scatter plot figures showed the percentages of CD4-1^+^ T cells among lymphocytes or T cells of isotype control and CD4-1-depleted tilapia, n = 3. These experiments were repeated for two independent times. *: *p*<0.05, **: *p*<0.01, determined by a two-tailed Student’s t-test.
(PDF)

S3 Fig Tilapia encode conserved transcription factors for Th1 differentiation. (**A**) Domain prediction of tilapia T-bet, STAT1 and STAT4. (**B-D**) Comparison of the domain organization of T-bet (**B**), STAT1 (**C**) and STAT4 (**D**) in tilapia and mouse. (**E-G**) Prediction of tertiary structures of T-bet (**E**), STAT1 (**F**) and STAT4 (**G**) from tilapia and mouse by SWISS-MODEL. The accession numbers of selected sequences were listed in S1 Table.
(PDF)

S4 Fig STAT1/T-bet axis is crucial for tilapia Th1 cell differentiation. (**A, B**) Spleen leukocytes were stimulated with P+I (**A**) or αCD3ε/CD28 (**B**) for indicated time. Relative mRNA levels of T-bet, STAT1 and STAT4 were examined by qPCR, n = 4–6. (**C**) Flow cytometry showed the phosphorylation level of STAT1 in lymphocytes that stimulated with P+I. (**D**) Tilapia was injected with STAT1 inhibitor for 2 days before spleen leukocytes were stimulated with PHA or CD3 mAb for 12 h. The expression level of T-bet was examined by qPCR, n = 6. (**E, F**) Spleen leukocytes from tilapia that *i*.*p*. injected with T-bet-specific, STAT1-specific or control siRNA for 2 days were harvested and stimulated with CD3ε mAb for 12 h. Relative mRNA levels of STAT4 were examined by qPCR, n = 4. (**G-J**) Tilapia *i*.*p*. injected with T-bet-specific, STAT1-specific or control siRNA were infected with *E*. *piscicida*, and spleen leukocytes were harvest for assay. Relative mRNA levels of indicated molecules (**G, H**), and percentages of CD3^+^CD4-1^+^ T cells **(I)** and CD3^+^CD4-1^+^IFN-γ^+^ T cells **(J)** on 5 DPI were examined, n = 4. (**K**) Tilapia individuals that infected with *E*. *piscicida* were injected with STAT1 inhibitor Fludarabine or PBS, and *E*. *piscicida* titers in liver were examined on 5 DPI, n = 5. These experiments were repeated for at least two independent times. *: *p*<0.05, **: *p*<0.01, ***: *p*<0.001, determined by a two-tailed Student’s t-test.
(PDF)

S5 Fig IL-12R and Runx3 expression during T-cell activation of Nile tilapia. Spleen leukcoytes were stimulated with P+I or PHA for indicated time, or with CD3ε/CD28 mAbs for 12 h. The mRNA levels of IL-12Rβ1, IL-12Rβ2 (**A**, **B**) or Runx3 (**C**) were examined by qPCR, n = 6. These experiments were repeated for three independent times. *: *p*<0.05, **: *p*<0.01, ***: *p*<0.001, determined by a two-tailed Student’s t-test.
(PDF)

S6 Fig Evolutionary properties of the Nile tilapia IL-2. (**A**) Comparison of IL-2 gene structure among Nile tilapia and vertebrates. Columns represent exons, and lines represent intron. (**B**) Domain prediction of IL-2 in indicated vertebrates. (**C**) Prediction of tertiary structures of IL-2 from indicated vertebrates by SWISS-MODEL. Blue: α-helix, purple: β-sheet, brown: coil. (**D**) Phylogenetic tree constructed with the amino acid sequences of IL-2 from the indicated species. The tree was constructed in MEGA7 by using neighbor-joining (NJ) method with 1000 bootstrap replications. The accession numbers of selected sequences were listed in S1 Table.
(PDF)

S7 Fig Structure and phylogenetic properties of IFN-γ receptors and STAT4 expression during RNAi. (**A**) Predicted tertiary structures of IFNγR1 and IFNγR2 from tilapia and mouse by SWISS-MODEL. (**B**) Phylogenetic tree constructed with the amino acid sequences of IFNγR1 and IFNγR2 from the indicated species. Phylogenetic tree was constructed in MEGA7 by using neighbor-joining (NJ) method with 1000 bootstrap replications. The accession numbers of selected sequences were listed in S1 Table. (**C, D**) Spleen leukocytes from tilapia that *i*.*p*. injected with T-bet-specific, STAT1-specific or control siRNA for 2 days were harvested and stimulated with recombinant IFN-γ for 12 h. Relative mRNA levels of STAT4 were examined by qPCR, n = 5. These experiments were repeated for two independent times.
(PDF)

S1 Table Information of genes used in present study. (PDF)

S2 Table Information and sequence of the primers used in present study. (PDF)
