# Supplementary figures and images for: IL-2–mTORC1 signaling coordinates the STAT1/T-bet axis to ensure Th1 cell differentiation and anti-bacterial immune response in fish
Source: PLoS Pathog. 2022 Oct 25;18(10):e1010913. doi: 10.1371/journal.ppat.1010913 (PMC9595569; doi:10.1371/journal.ppat.1010913)

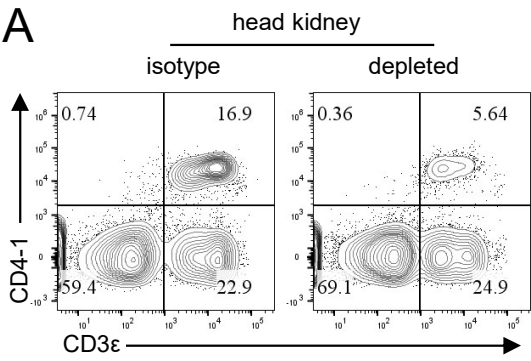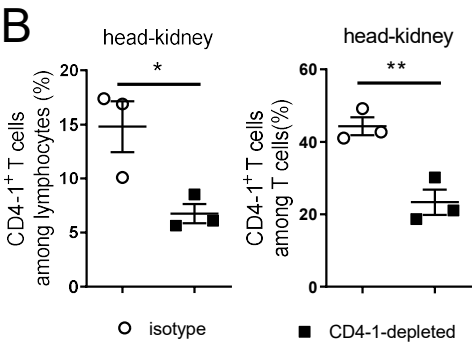

Supplement: S2 Fig — CD4-1+ cells were depleted as described in Fig 3F. (A) Flow cytometry showed the percentage of CD3+CD4-1+ T cells in head kidney lymphocytes of CD4-1-depleted or non-depleted tilapia on 9-day post depletion. (B) Scatter plot figures showed the percentages of CD4-1+ T cells among lymphocytes or T cells of isotype control and CD4-1-depleted tilapia, n = 3. These experiments were repeated for two independent times. *: p<0.05, **: p<0.01, determined by a two-tailed Student’s t-test. (PDF) [file ppat.1010913.s002.pdf]

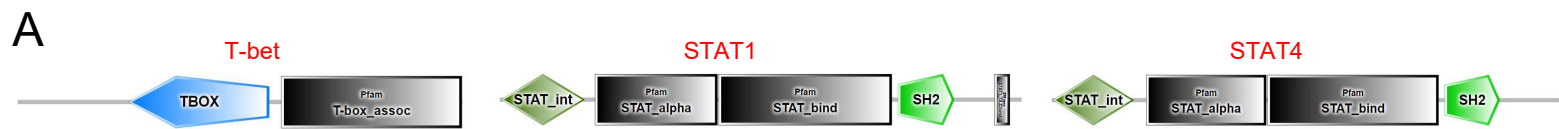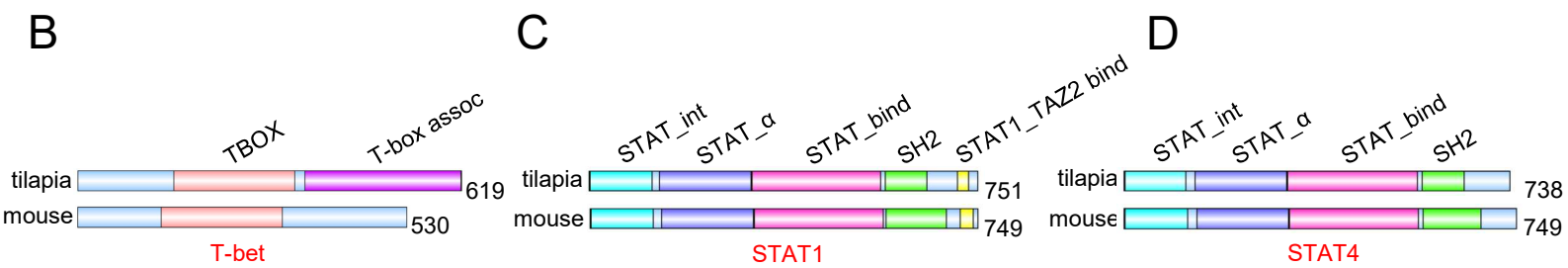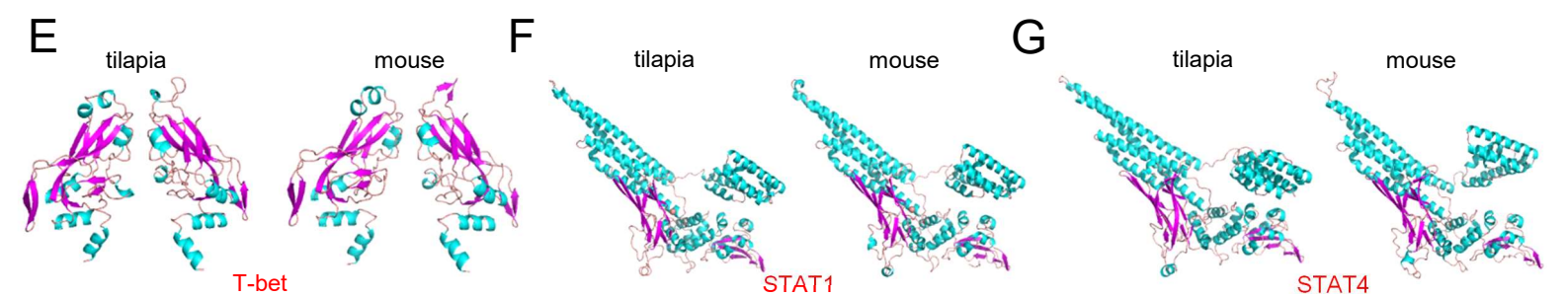

Supplement: S3 Fig — (A) Domain prediction of tilapia T-bet, STAT1 and STAT4. (B-D) Comparison of the domain organization of T-bet (B), STAT1 (C) and STAT4 (D) in tilapia and mouse. (E-G) Prediction of tertiary structures of T-bet (E), STAT1 (F) and STAT4 (G) from tilapia and mouse by SWISS-MODEL. The accession numbers of selected sequences were listed in S1 Table. (PDF) [file ppat.1010913.s003.pdf]

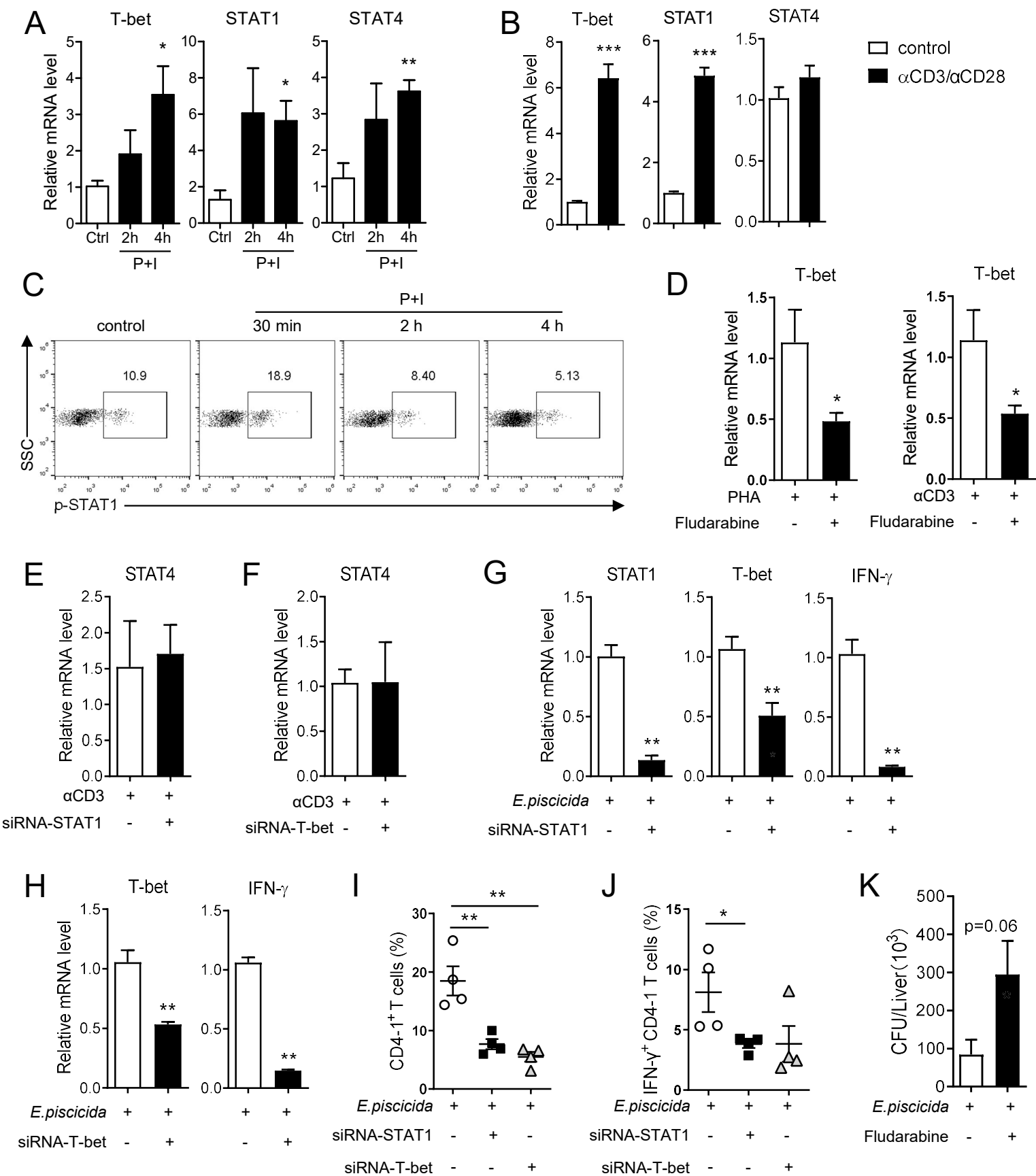

Supplement: S4 Fig — (A, B) Spleen leukocytes were stimulated with P+I (A) or αCD3ε/CD28 (B) for indicated time. Relative mRNA levels of T-bet, STAT1 and STAT4 were examined by qPCR, n = 4–6. (C) Flow cytometry showed the phosphorylation level of STAT1 in lymphocytes that stimulated with P+I. (D) Tilapia was injected with STAT1 inhibitor for 2 days before spleen leukocytes were stimulated with PHA or CD3 mAb for 12 h. The expression level of T-bet was examined by qPCR, n = 6. (E, F) Spleen leukocytes from tilapia that i.p. injected with T-bet-specific, STAT1-specific or control siRNA for 2 days were harvested and stimulated with CD3ε mAb for 12 h. Relative mRNA levels of STAT4 were examined by qPCR, n = 4. (G-J) Tilapia i.p. injected with T-bet-specific, STAT1-specific or control siRNA were infected with E. piscicida, and spleen leukocytes were harvest for assay. Relative mRNA levels of indicated molecules (G, H), and percentages of CD3+CD4-1+ T cells (I) and CD3+CD4-1+IFN-γ+ T cells (J) on 5 DPI were examined, n = 4. (K) Tilapia individuals that infected with E. piscicida were injected with STAT1 inhibitor Fludarabine or PBS, and E. piscicida titers in liver were examined on 5 DPI, n = 5. These experiments were repeated for at least two independent times. *: p<0.05, **: p<0.01, ***: p<0.001, determined by a two-tailed Student’s t-test. (PDF) [file ppat.1010913.s004.pdf]

**A**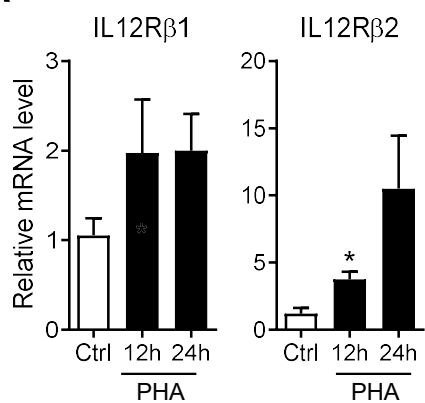**B**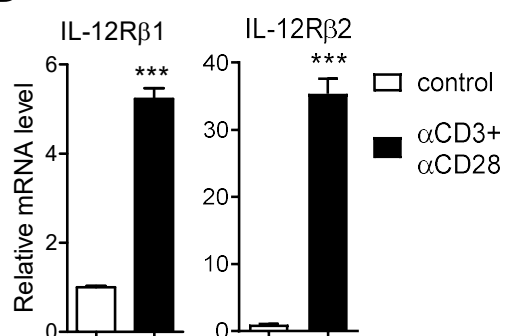**C**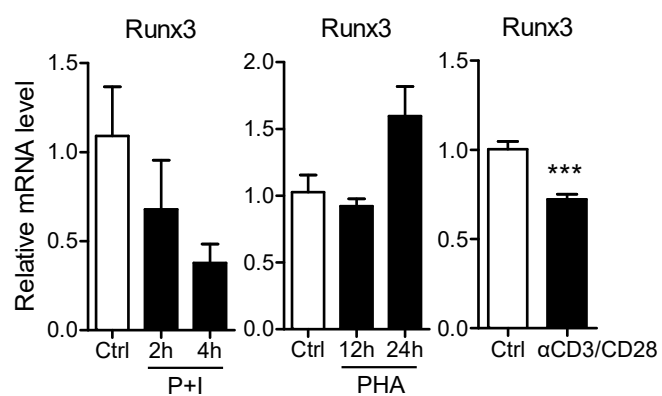

Supplement: S5 Fig — Spleen leukcoytes were stimulated with P+I or PHA for indicated time, or with CD3ε/CD28 mAbs for 12 h. The mRNA levels of IL-12Rβ1, IL-12Rβ2 (A, B) or Runx3 (C) were examined by qPCR, n = 6. These experiments were repeated for three independent times. *: p<0.05, **: p<0.01, ***: p<0.001, determined by a two-tailed Student’s t-test. (PDF) [file ppat.1010913.s005.pdf]

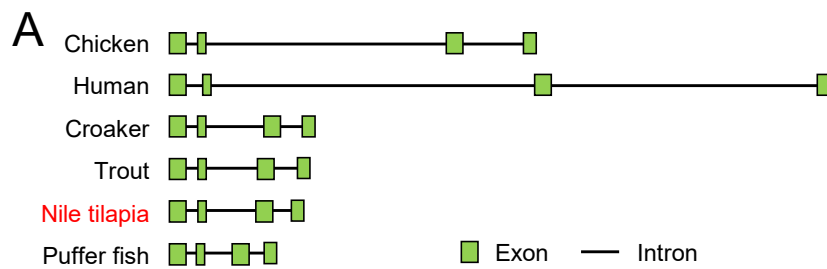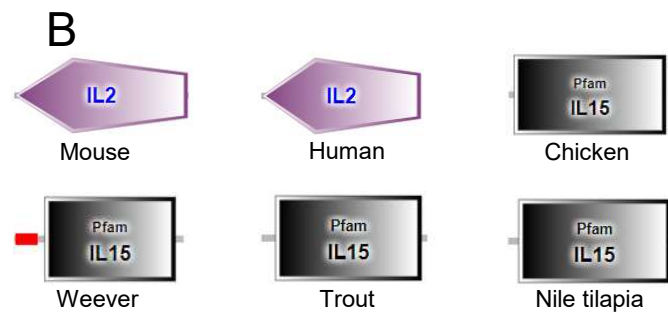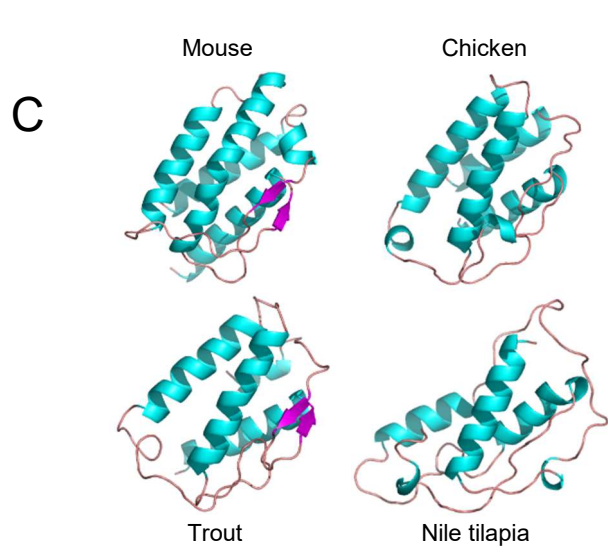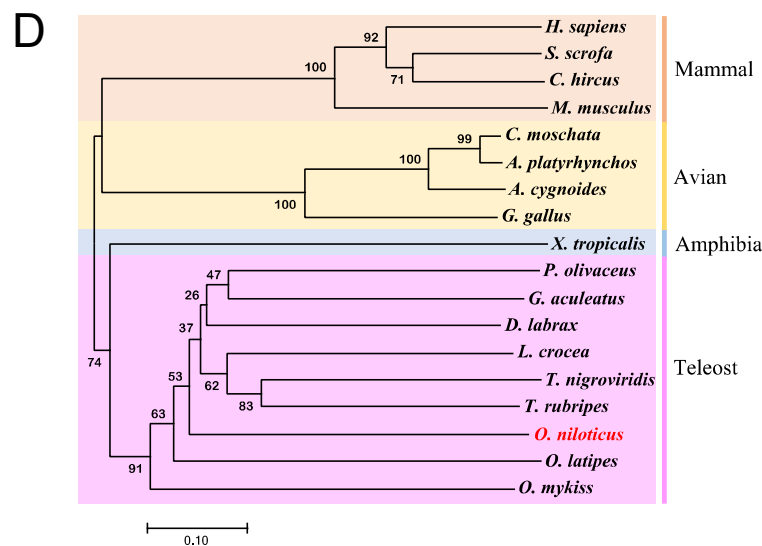

Supplement: S6 Fig — (A) Comparison of IL-2 gene structure among Nile tilapia and vertebrates. Columns represent exons, and lines represent intron. (B) Domain prediction of IL-2 in indicated vertebrates. (C) Prediction of tertiary structures of IL-2 from indicated vertebrates by SWISS-MODEL. Blue: α-helix, purple: β-sheet, brown: coil. (D) Phylogenetic tree constructed with the amino acid sequences of IL-2 from the indicated species. The tree was constructed in MEGA7 by using neighbor-joining (NJ) method with 1000 bootstrap replications. The accession numbers of selected sequences were listed in S1 Table. (PDF) [file ppat.1010913.s006.pdf]

A

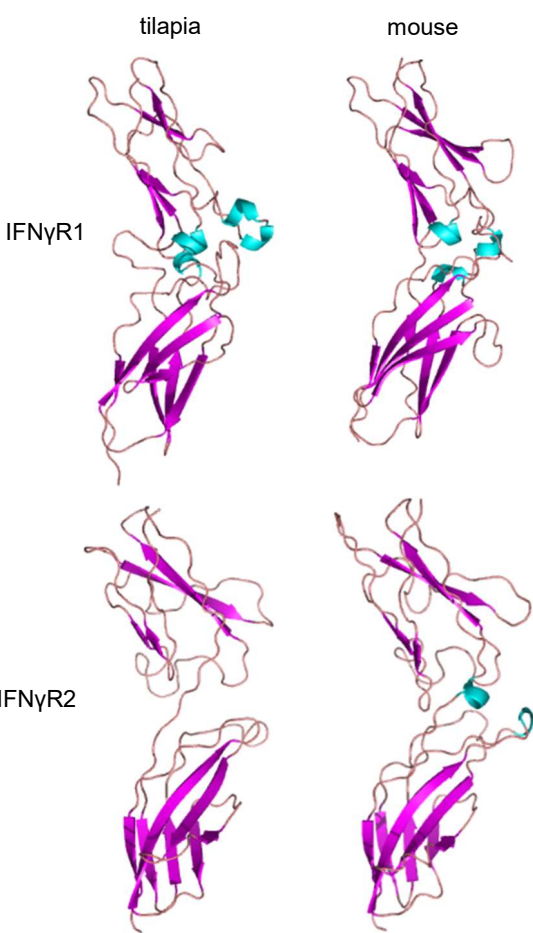

B

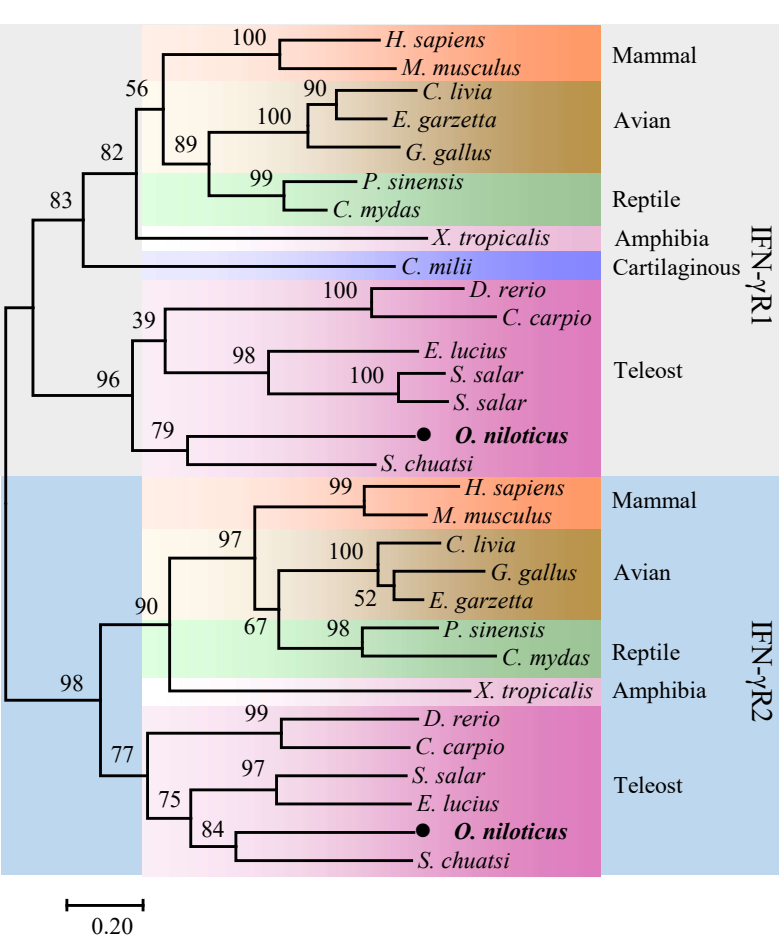

C

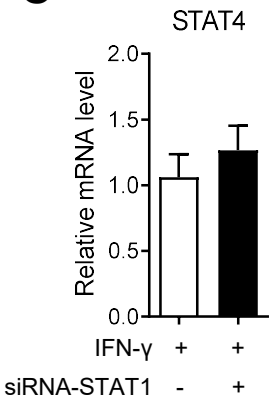

D

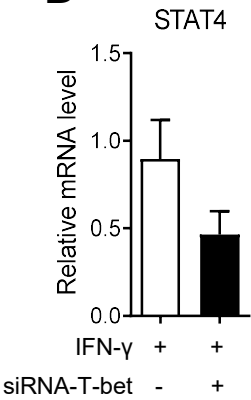

Supplement: S7 Fig — (A) Predicted tertiary structures of IFNγR1 and IFNγR2 from tilapia and mouse by SWISS-MODEL. (B) Phylogenetic tree constructed with the amino acid sequences of IFNγR1 and IFNγR2 from the indicated species. Phylogenetic tree was constructed in MEGA7 by using neighbor-joining (NJ) method with 1000 bootstrap replications. The accession numbers of selected sequences were listed in S1 Table. (C, D) Spleen leukocytes from tilapia that i.p. injected with T-bet-specific, STAT1-specific or control siRNA for 2 days were harvested and stimulated with recombinant IFN-γ for 12 h. Relative mRNA levels of STAT4 were examined by qPCR, n = 5. These experiments were repeated for two independent times. (PDF) [file ppat.1010913.s007.pdf]
