# Supplementary material for: IL-2–mTORC1 signaling coordinates the STAT1/T-bet axis to ensure Th1 cell differentiation and anti-bacterial immune response in fish
Source: PLoS Pathog. 2022 Oct 25;18(10):e1010913. doi: 10.1371/journal.ppat.1010913 (PMC9595569; doi:10.1371/journal.ppat.1010913)
Supplement: S1 Table — (PDF) [file ppat.1010913.s008.pdf]

S1 Table. Information of genes used in present study

Genes	Accession No.	Species	Application
IFN-γ	AAB59534.1	*Homo sapiens*	MSA, PA
IFN-γ	NP_990480.1	*Gallus gallus*	MSA, PA
IFN-γ	NP_001273831.1	*Pelodiscus sinensis*	MSA, PA
IFN-γ	XP_018107195.1	*Xenopus laevis*	MSA, PA
IFN-γ	BAD06253.1	*Danio rerio*	MSA, PA
IFN-γ related	NP_001018629.1	*Danio rerio*	MSA, PA
IFN-γ	P30123.1	*Oryctolagus cuniculus*	PA
IFN-γ	EDL24379.1	*Mus musculus*	PA
IFN-γ	ABF18829.1	*Columba livia*	PA
IFN-γ	XP_007065892.1	*Chelonia mydas*	PA
IFN-γ	XP_002938555.1	*Xenopus tropicalis*	PA
IFN-γ	PIO34111.1	*Lithobates catesbeianus*	PA
IFN-γ	XP_007903185.1	*Callorhinchus milii*	PA
IFN-γ	XP_020383749.1	*Rhincodon typus*	PA
IFN-γ	QBC17796.1	*Acipenser sinensis*	PA
IFN-γ	XP_033887986.1	*Acipenser ruthenus*	PA
IFN-γ like	XP_033881006.1	*Acipenser ruthenus*	PA
IFN-γ	XP_015208415.1	*Lepisosteus oculatus*	PA
IFN-γ	XP_036375558.1	*Megalops cyprinoides*	PA
IFN-γ like	XP_036375559.1	*Megalops cyprinoides*	PA
IFN-γ	QDO15115.1	*Siniperca chuatsi*	PA
IFN-γ-like	QDO15116.1	*Siniperca chuatsi*	PA
IFN-γ related	ACN56579.1	*Ctenopharyngodon idella*	PA
IFN-γ	AGQ16237.1	*Ctenopharyngodon idella*	PA
IFN-γ related	NP_001347641.1	*Cyprinus carpio*	PA
IFN-γ	NP_001348151.1	*Cyprinus carpio*	PA
IFN-γ2	NP_001153976.1	*Oncorhynchus mykiss*	PA
IFN-γ	CAE82300.1	*Oncorhynchus mykiss*	PA
IFN-γ	ACN37863.1	*Salmo salar*	PA
IFN-γ	AAZ40505.1	*Ictalurus punctatus*	PA
IFN-γ related	NP_001187146.1	*Ictalurus punctatus*	PA
IFN-γ	AFM31242.1	*Epinephelus coioides*	PA
IFN-γ1	QEA72089.1	*Epinephelus coioides*	PA
IFN-γ	AHZ62713.1	*Tetraodon nigroviridis*	PA
IFN-γ related	AHZ62714.1	*Tetraodon nigroviridis*	PA

IFN-γ	XP_034535351.1	*Notolabrus celidotus*	PA
IFN-γ like	XP_034535345.1	*Notolabrus celidotus*	PA
IFN-γ like	XP_029131411.1	*Labrus bergylta*	PA
IFN-γ	NP_001347671.1	*Esox lucius*	PA
IFN-γ related	XP_026779533.1	*Pangasianodon hypophthalmus*	PA
IFN-γ	XP_026779390.1	*Pangasianodon hypophthalmus*	PA
T-bet	NP_037483.1	*Homo sapiens*	MSA
T-bet	NP_062380.2	*Mus musculus*	3D
T-bet	XP_015154966.3	*Gallus gallus*	MSA
T-bet	XP_037743046.1	*Chelonia mydas*	MSA
T-bet	XP_002940203.2	*Xenopus tropicalis*	MSA
T-bet	XP_003448706.2	*Oreochromis niloticus*	MSA, 3D
T-bet	NP_001164070.1	*Danio rerio*	MSA
STAT1	XP_019207486.1	*Oreochromis niloticus*	MSA, 3D
STAT1	AAA19454.1	*Mus musculus*	3D
STAT1	ADA59516.1	*Homo sapiens*	MSA
STAT1	NP_001012932.1	*Gallus gallus*	MSA
STAT1	XP_012826050.2	*Xenopus tropicalis*	MSA
STAT4	AAA19453.1	*Mus musculus*	3D
STAT4	NP_001230764.1	*Homo sapiens*	MSA
STAT4	NP_001254484.2	*Gallus gallus*	MSA
STAT4	XP_031749081.1	*Xenopus tropicalis*	MSA
STAT4	XP_013121682.1	*Oreochromis niloticus*	MSA, 3D
CD4-1	ENSONIP00000016379.2	*Oreochromis niloticus*	3D
IL-2	NG_016779	*Homo sapiens*	PA, 3D
IL-2	NP_032392.1	*Mus musculus*	PA, 3D
IL-2	NC_006091	*Gallus gallus*	PA, 3D
IL-2	ENSONIT00000028270.2	*Oreochromis niloticus*	PA, 3D
IL-2	AIK66531.1	*Dicentrarchus labrax*	PA, 3D
IL-2	NP_001157537.1	*Oncorhynchus mykiss*	PA, 3D
IL-2	ACT78884.1	*Sus scrofa*	PA
IL-2	ABN51231.1	*Capra hircus*	PA
IL-2	AAO39413.1	*Cairina moschata*	PA
IL-2	AFR11500.1	*Anser cygnoides*	PA
IL-2	NP_001297302.1	*Anas platyrhynchos*	PA
IL-2	ABS44962.1	*Xenopus tropicalis*	PA
IL-2	ARD05071.1	*Larimichthys crocea*	PA

IL-2	ABS44960.1	*Tetraodon nigroviridis*	PA
IL-2	NP_001254611.1	*Gasterosteus aculeatus*	PA
IL-2	NP_001033083.1	*Takifugu rubripes*	PA
IL-2	ABS44958.1	*Oryzias latipes*	PA
IL-2	BCL05865.1	*Paralichthys olivaceus*	PA
IFN-γR1	NP_001347779.1	*Oreochromis niloticus*	MSA, 3D, PA
IFN-γR1	NP_034641.1	*Mus musculus*	MSA, 3D, PA
IFN-γR1	AAH05333.1	*Homo sapiens*	MSA, PA
IFN-γR1	NP_001123859.1	*Gallus gallus*	MSA, PA
IFN-γR1	NP_001072814.2	*Xenopus tropicalis*	MSA, PA
IFN-γR1	NP_001129451.2	*Danio rerio*	MSA, PA
IFN-γR1	PKK32935.1	*Columba livia*	PA
IFN-γR1	XP_035755794.1	*Egretta garzetta*	PA
IFN-γR1	XP_006112786.2	*Pelodiscus sinensis*	PA
IFN-γR1	XP_007058131.2	*Chelonia mydas*	PA
IFN-γR1	XP_044045456.1	*Siniperca chuatsi*	PA
IFN-γR1	NP_001348131.2	*Cyprinus carpio*	PA
IFN-γR1a	NP_001347848.1	*Salmo salar*	PA
IFN-γR1b	NP_001347849.1	*Salmo salar*	PA
IFN-γR1	XP_034150384.1	*Esox lucius*	PA
IFN-γR1	XP_007891179.1	*Callorhinchus milii*	PA
IFN-γR2	NP_001348024.1	*Oreochromis niloticus*	MSA, 3D, PA
IFN-γR2	NP_032364.1	*Mus musculus*	PA, 3D
IFN-γR2	NP_001008676.1	*Gallus gallus*	MSA, PA
IFN-γR2	AAH03624.1	*Homo sapiens*	MSA, PA
IFN-γR2	XP_025041893.1	*Pelodiscus sinensis*	MSA, PA
IFN-γR2	XP_004912198.1	*Xenopus tropicalis*	MSA, PA
IFN-γR2	NP_001071095.2	*Danio rerio*	MSA, PA
IFN-γR2	PKK32617.1	*Columba livia*	PA
IFN-γR2	XP_009639266.2	*Egretta garzetta*	PA
IFN-γR2	XP_027681846.1	*Chelonia mydas*	PA
IFN-γR2	NP_001347698.1	*Cyprinus carpio*	PA
IFN-γR2	NP_001348050.1	*Salmo salar*	PA
IFN-γR2	NP_001347680.1	*Esox lucius*	PA
IFN-γR2	XP_044071627.1	*Siniperca chuatsi*	PA

Note:

MSA: multiple sequence alignment; PA: phylogenetic analysis; 3D: 3D structure analysis.
