# Supplementary material for: IL-2–mTORC1 signaling coordinates the STAT1/T-bet axis to ensure Th1 cell differentiation and anti-bacterial immune response in fish
Source: PLoS Pathog. 2022 Oct 25;18(10):e1010913. doi: 10.1371/journal.ppat.1010913 (PMC9595569; doi:10.1371/journal.ppat.1010913)
Supplement: S2 Table — (PDF) [file ppat.1010913.s009.pdf]

S2 Table. Information and sequence of the primers used in present study

Genes	Accession No.	Direction	Sequence
β-actin	KJ126772.1	Forward	CGGAATCCACGAAACCACCTA
		Reverse	CCAGACGGAGTATTTACGCTCA
IFN-γ	NM_001287402.1	Forward	GGGTGGTGTTTTGGAGTCGT
		Reverse	GTAGCGAGCCTGAGTTGTTGGTG
CD4-1	ENSONIT00000016394	Forward	CCAAGGGAAACAGAGAAGGAAA
		Reverse	AAGGGATGGTGAGAGGTGAAAC
CD8α	ENSONIT00000025699.1	Forward	CATAACAGCAAAGGAAGGACAG
		Reverse	TACCTTGGATAAGTGACGCA
CD3ε	XM_003449297.5	Forward	CTGGAGGACCAAAGTGACG
		Reverse	CACGCATTTCCTTCAACA
TCRβ	HM162889.1	Forward	TTCTACCCTGACCATGTCAAAA
		Reverse	TTCCCATTGTTTGGCATAGAC
IgM	KC677037.1	Forward	TGGCTTGTGGATGACGAGGA
		Reverse	AGCACTTGGAGTCTTGGTTGATG
CD20	XM_019364882.2	Forward	TCGGAGCAATCCTTGTAAGCAGA
		Reverse	GGCAAAAGCAACCCCAGTCA
IFNγR1	NM_001360850.1	Forward	ATGTAAAGGTCTCTGTCCGCT
		Reverse	GTTGCTCAGGTCATACTCGTG
IFNγR2	NM_001361095.1	Forward	AGGAACACGCCGAGAATG
		Reverse	AGGGATGACCTCACACTGGA
T-bet	XM_003448658.4	Forward	ACCTCGGTCACCCAACTAAATC
		Reverse	CACCCACACCTCCCTCAAAT
Stat1	XM_019351941.2	Forward	TCCACGACCTTCTCGCTCA
		Reverse	TCTCATCCTCGGTGCTCTTTG
Stat4	XM_013266228.3	Forward	GGCGATAACGGTGAGGTGAA
		Reverse	GGGTTGATGGGATGTAAGGATG
IL-2	ENSONIT00000028270.2	Forward	ATGTCGAGACCCAGGGAAAC
		Reverse	CAGGCCACAGGTGACAGTTA
CD122	XM_019360337.2	Forward	AGCCAGATGAGAAGATGATGTTGAA
		Reverse	TGAGTGAGGAAATAGGAGGAGGG
IL-12Rβ1	XM_005453984.4	Forward	TTCGGCTTGCTGTTTGTCTTC
		Reverse	AGTCTTTGATTGATGGGGGATG
IL-12Rβ2	XM_025903204.1	Forward	GGGCTGTGGTGGCAAAAGTT
		Reverse	TTGGAGTAGAGCAGTATGGGTGA
Human-β-actin	JN038572.1	Forward	ATCGTGCGTGACATCAAAGAGA
		Reverse	CAGGAAGCAAGGCTGGAAGA
Human-IFN-γ	NM_000619.3	Forward	TGGAGACCATCAAGGAAGACA
		Reverse	GCGACAGTTCAGCCATCAC
rIFN-γ	NM_001287402.1	Forward	CGGAATTCTCCCACATCCCAGCAGAGA
		Reverse	CCCTCGAGTTAAACTCTGGGGCGACCTTT
rIFNγR1	NM_001360850.1	Forward	CGGGATCCGTGAGTGTTTCACCTCCAA

		Reverse	CCCTCGAGCTCATTGAAACCTGGGTAT
rIFNγR2	NM_001361095.1	Forward	CGGGATCCATGCTGCTGATCCTGCTCTG
		Reverse	CCCTCGAGCATCGGCGTCCTGTTCAC
rIL-2	ENSONIT00000028270.	Forward	CCCTCGAGCAAAACCTATATCCAAAC
		Reverse	GAAGATCTGACTTCCGTGTTTATTTTG
rStat1	XM_019351941.2	Forward	CGGGATCCGCCACCATGGCGCAGTGGGGCCAG
		Reverse	CCAAGCTTTCAGTTTCCATCTCGAAAGTCT
rT-bet	XM_003448658.4	Forward	CGGAATTCGCCACCATGGGCGGCATAGGTGGCA
		Reverse	CAAGCTTTCAGTGGGTGTAATAACCATAA
rStat4	XM_013266228.3	Forward	CGGGATCCGCCACCATGAGCCAGTGGAAGCA
		Reverse	CGGAATTCCTCTGAATACGGAGAGCT
rIFNγ-promoter	CM007498.2	Forward	GGGGTACCTTTTGCCTAATGTTCTGTAT
		Reverse	CCCTCGAGGTAGTTGAGTCTTCGGTTTG
siRNA-STAT1-1	XM_019351941.2	Forward	GCUGCAUCAUGGGAUUCAUTT
		Reverse	AUGAAUCCCAUGAUGCAGCTT
siRNA-STAT1-2	XM_019351941.2	Forward	GGCCCUGUUUGAUAAGGAUTT
		Reverse	AUCCUUAUCAAACAGGGCCTT
siRNA-STAT1-3	XM_019351941.2	Forward	GGAGCUCAUUUCGGUUUCATT
		Reverse	UGAAACCGAAAUGAGCUCCTT
siRNA-T-bet-1	XM_003448658.4	Forward	GGGUGGUUUAGACCCAUAUTT
		Reverse	AUAUGGGUCUAAACCACCCTT
siRNA-T-bet-2	XM_003448658.4	Forward	CAGCGAUGCAGACUAUUAUTT
		Reverse	AUAAUAGUCUGCAUCGCUGTT
siRNA-T-bet-3	XM_003448658.4	Forward	GGAUCCUACUGCCCAUUAUTT
		Reverse	AUAAUGGGCAGUAGGAUCCTT
control siRNA		Forward	UUCUCCGAACGUGUCACGUTT
		Reverse	ACGUGACACGUUCGGAGAA TT
