# Supplementary material for: IL-2–mTORC1 signaling coordinates the STAT1/T-bet axis to ensure Th1 cell differentiation and anti-bacterial immune response in fish
Source: PLoS Pathog. 2022 Oct 25;18(10):e1010913. doi: 10.1371/journal.ppat.1010913 (PMC9595569; doi:10.1371/journal.ppat.1010913)
Supplement: S1 Fig — (A) The synteny analysis of tilapia IFN-γ and its homologues from other vertebrates. (B) Gene structures of tilapia IFN-γ and its vertebrate counterparts. (C) Multiple sequence alignment of IFN-γ from tilapia and other vertebrates. Amino acid residues with 60% identity are in black, and similar residues are in dark gray. Some important regions were marked out, with underline for signal peptide, with overline for NLS. The sequence of signature motif ([I/V]-Q-X-[K/Q]-A-X2-E-[L/F]-X2-[I/V]) was boxed. (D) Phylogenetic tree constructed with the amino acid sequences of IFN-γ from the indicated species. Phylogenetic tree was constructed in MEGA7 by using neighbor-joining (NJ) method with 1000 bootstrap replications. The accession numbers of selected sequences were listed in S1 Table. (PDF) [file ppat.1010913.s001.pdf]

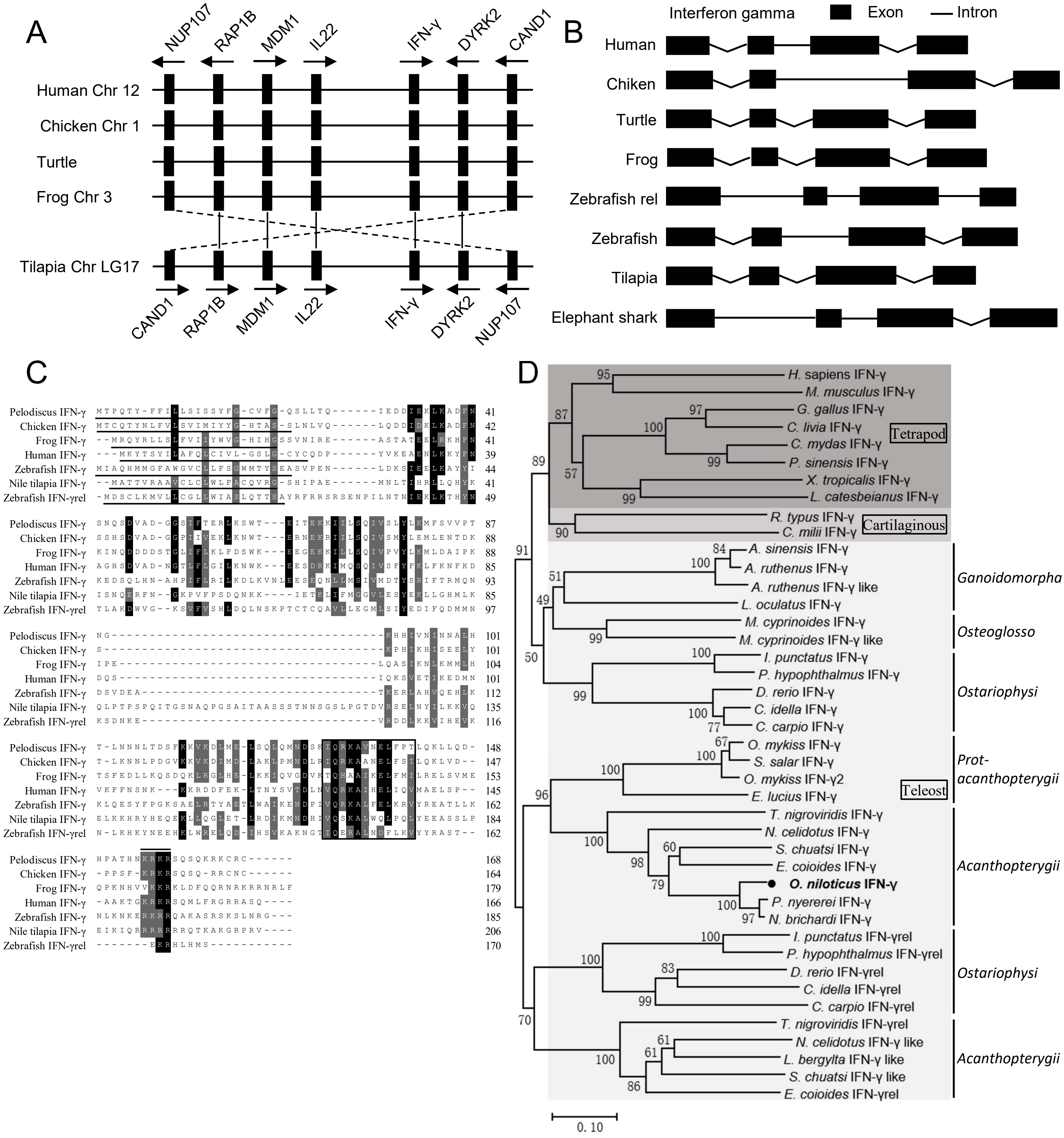
A
NUP107
RAP1B
MDM1
IL22
IFN-γ
DYRK2
CAND1
Human Chr 12
Chicken Chr 1
Turtle
Frog Chr 3
Tilapia Chr LG17
B
Interferon gamma
Exon
Intron
Human
Chiken
Turtle
Frog
Zebrafish rel
Zebrafish
Tilapia
Elephant shark
C
Pelodiscus IFN-γ
Chicken IFN-γ
Frog IFN-γ
Human IFN-γ
Zebrafish IFN-γ
Nile tilapia IFN-γ
Zebrafish IFN-γrel
D
H. sapiens IFN-γ
M. musculus IFN-γ
G. gallus IFN-γ
C. livia IFN-γ
C. mydas IFN-γ
P. sinensis IFN-γ
X. tropicalis IFN-γ
L. catesbeianus IFN-γ
Tetrapod
R. typus IFN-γ
C. milii IFN-γ
Cartilaginous
A. sinensis IFN-γ
A. ruthenus IFN-γ
A. ruthenus IFN-γ like
L. oculatus IFN-γ
Ganoidomorpha
M. cyprinoides IFN-γ
M. cyprinoides IFN-γ like
Osteoglosso
I. punctatus IFN-γ
P. hypophthalmus IFN-γ
D. rerio IFN-γ
C. idella IFN-γ
C. carpio IFN-γ
Ostariophysi
O. mykiss IFN-γ
S. salar IFN-γ
O. mykiss IFN-γ2
E. lucius IFN-γ
Prot-acanthopterygii
Teleost
T. nigroviridis IFN-γ
N. celidotus IFN-γ
S. chuatsi IFN-γ
E. coioides IFN-γ
O. niloticus IFN-γ
P. nyererei IFN-γ
N. brichardi IFN-γ
Acanthopterygii
I. punctatus IFN-γrel
P. hypophthalmus IFN-γrel
D. rerio IFN-γrel
C. idella IFN-γrel
C. carpio IFN-γrel
Ostariophysi
T. nigroviridis IFN-γrel
N. celidotus IFN-γ like
L. bergylta IFN-γ like
S. chuatsi IFN-γ like
E. coioides IFN-γrel
Acanthopterygii
0. 10
